# Chatting with an LLM-based AI elicits affective and cognitive processes in education for sustainable development

**DOI:** 10.1038/s41598-026-39317-6

**Published:** 2026-02-21

**Authors:** Pia Spangenberger, Georg Felix Reuth, Jule M. Krüger, Lena Baumann, Steve Nebel

**Affiliations:** Department of Educational Sciences, University of Potsdam, Potsdam, Germany

**Keywords:** Environmental social sciences, Psychology and behaviour, Computer science

## Abstract

Personalized interactions have been discussed as beneficial for learning for decades. Now, with the rise of generative artificial intelligence (GenAI), personalized artificial human-like conversations may impact the quality of learning. Manipulating system prompts to design personalities has the potential to enhance the quality of conversation with Large Language Model (LLM)-based AI. However, it is still uncertain exactly to what extent the emotional tone of a generative AI chatbot is relevant for learning. Hence, the current study evaluates the impact of a chat-based conversation with an LLM-based AI on relevant affective (empathy, compassion, distress) and cognitive (perspective-taking, reflection, knowledge) processes in education for sustainable development. Here, the focus is on both the general impact and the particular impact of two different system prompts that assign the AI’s specific personality traits (empathic vs. compassionate). Comparing these two groups and one control group reading a text (*N* = 122) indicates that chatting with an empathic AI can elicit stronger emotions (e.g., empathy, compassion, distress) compared to chatting with a compassionate AI, and compared to the control. Although all groups gained knowledge, we found no group differences. Further research is necessary to ensure reliable and contextually appropriate conversations in the context of education.

**Supplementary Information:**

The online version contains supplementary material available at 10.1038/s41598-026-39317-6.

## Introduction

### Main

Research on artificial intelligence (AI) has come a long way since 1956, when “a group of scientists made it the subject of a specific event: the Dartmouth Summer Research Project on Artificial Intelligence”^1^. Today, in times of an increasing use of Large Language Model (LLM)-based AIs^2^ as virtual assistants^3^, the use of LLM-based chatbots in the context of education is part of the debate^4,5^. Students can chat with adaptive AIs that provide personalized and interactive human-like conversations. While earlier virtual assistants like chatbots only provided linear, rule-based conversations, LLM-based chatbots, as technological systems that imitate human intelligence, can frame a conversation within a larger context and respond in a human-like way^1,6^. As already pointed out by Ji, Han, and Ko [6] , this is one of the main affordances of using today’s GenAI as an educational tool: the possibility of human-like communication. Furthermore, GenAI can impersonate individuals or entities with whom learners cannot usually talk, such as deceased individuals of historic relevance^7^, non-verbal entities, or objects such as a tree speaking for nature, which is the focus of the present study. For the purpose of this study, LLM-based chatbot refers to conversational systems powered by large language models.

In the context of education, empirical evidence supports the advantages of personal conversations over reading texts^8^. In line with the Personalization Principle of the Cognitive Theory of Multimedia Learning (CTML), personalization is one crucial component of learning with digital media^9^. Based on the process of self-referencing in information encoding^10^, instructional researchers revealed beneficial effects on learning when directly addressing learners or using informal language^11,12^. This effect was widely supported by meta-analytic evidence^8^, indicating further positive effects of the conversational style on cognitive processing, such as knowledge retention or the ability to apply knowledge. The Cognitive-Affective-Social Theory of Learning in Digital Environments (CASTLE), as an extension of CTML, furthermore describes the importance of social processes and cues for encouraging and supporting cognitive and affective processing when learning with digital materials^13^. In line with the theory of computers as social actors (CASA)^14,15^, learners can also build a personal emotional connection with a computer-supported system. These social responses can be reinforced by certain media affordances^16^. There is evidence that personalization of a social agent can evoke greater social responses^17^. Similarly, anthropomorphism, i.e. the attribution of human-like features, leads to faster processing and presumably plays a major role in the extent to which digital social agents can trigger social reactions^16^. These findings also apply to a text-based conversation with an LLM-based virtual assistant^18,19^, as already observed in the use of the first rule-based ELIZA chatbot in the 1960s, when “people naturally attribute intelligence to and anthropomorphize computational systems”^3^. Earlier studies have also shown that humans can treat a service chatbot similarly to a human, supporting the media equivalency hypothesis^20^. Although AIs are recognized as a technology, they can elicit social reactions, which, for instance, has also been observed in the use of smart speakers or robots^21,22^. Witnessing harm towards robots or smart speakers can induce empathy within observers^21–23^. Furthermore, the perceived personalization and anthropomorphism of an LLM-based conversational chatbot can increase the perceived warmth and competence of the AI, which enhances the perception that the AI is displaying empathy and, in turn, fosters users’ willingness to engage with it^24^. Communication with an LLM-based service chatbot can also induce so-called ‘connection emotions’ such as empathy, understanding, fairness, and friendliness^18^. A qualitative study revealed that a conversation with an LLM-based chatbot could induce children to share their personal emotions with it^25^. Another study, evaluating 160 conversations with one’s future self represented by an LLM-based chatbot compared to a rule-based chatbot^26^, could show that the LLM reduced participants’ negative emotions and anxiety in regard to the future. Hence, a conversation with an LLM-based chatbot therefore seems to be a promising approach to providing information and eliciting emotions.

Regarding learning, researchers recognize the importance of combining affective and cognitive processes (e.g., Plass & Kaplan^27^ ). As Plass and Kaplan^27^ explain in their Integrated Cognitive Affective Model of Learning with Multimedia (ICALM), emotional design elements can have a beneficial effect on important components of learning, such as attention and motivation. The above-mentioned CASTLE furthermore states that it is important to consider the effect of social processes and their interaction with affective and cognitive processes when designing digital learning materials^13^.

However, research in the context of education combining affect and cognitive processes and its impact on learning outcomes induced by a conversation with an LLM-based chatbot is still in the early stages^28^. A recent meta-analysis outlined the benefits of LLM-based chatbots for learning^29^, observing that ChatGPT can enhance learning performance, both improving learning perception and promoting higher-order thinking. A large effect was revealed for problem-based learning. However, all of the above effects might vary depending on the instructional approach and the duration of the intervention^29^. The personalized learning experience and the deeper engagement with the material have been identified as some of the unique affordances of such LLM-based conversations, as outlined in the review by Celik et al.^30^. In particular, perceiving personalized feedback and a deeper engagement with the learning content during a conversation have been found as some of the most promising affordances of GenAI-based tools for learning, whereas chatbots have been assessed as particularly well-suited to foster communication, problem-based learning, and critical thinking skills. However, affective processes initiated by GenAI chatbots and their impact on learning have rarely been investigated as of yet, as noted by Yin et al.^31^, stating that “students’ behavioural engagement by ambivalent emotions remains uncertain within the context of educational chatbot interactions (Lai et al., 2021; Tze et al., 2022)” (p.4), and pointing out that “educators could enhance the learning experience with anthropomorphic emotional cues.  . . . [to utilize] personalized characteristics of chatbots” (p. 19).

Regarding affective processes, in an essay-writing task supported by LLM-based feedback, an increase in positive emotions, such as curiosity, enjoyment, and pride, has been observed^32^. In regard to a problem-solving task, the use of a ChatGPT could induce self-efficacy and enhance the performance quality and originality of students’ ideas^33^. A systematic review on affective, cognitive, and behavioral learning outcomes in the domain of English language teaching^34^ found that the use of conversational AI-based tools could promote enjoyment, interest, and motivation in the affective domain and improve speaking skills in the cognitive domain. Comparing ChatGPT to a Google search engine within a problem-solving task, it was also observed that the use of the GenAI could lower the cognitive load of students, but the search engine group produced higher-quality arguments^35^. Hence, with the rapid development of new LLMs, further research is needed to evaluate the potential for combining affective and cognitive learning processes. Consequently, in the current study, we focus on the affordances of a generative chatbot’s personal characteristics by manipulating its emotional tone to investigate how it impacts relevant cognitive and affective processes for learning in education for sustainable development.

### Affective and cognitive processes in education for sustainable development

Research in the field of education for sustainable development and environmental psychology postulates a stronger focus on combining cognitive (e.g., knowledge, perspective-taking, reflection) and affective processes (e.g., distress, nature connectedness) to induce change in learners^36–39^. For instance, nature connectedness, an affective bond with nature^40^, related to compassion^36^, has been shown to be a relevant feeling to foster pro-environmental behavior^41,42^. The underlying idea behind this approach is that people should be able to understand and acknowledge nature’s needs in order to take action. Affective processes that have been investigated in research on nature connectedness and education for sustainable development include distress, empathy, and compassion. Distress has been discussed in the context of message framing when communicating climate change issues^43^, and can play a role in inducing an urge to change in people when it comes to climate change action^38^. Empathy and compassion partially overlap in terms of content, but while empathy can also lead to stress, compassion is more associated with pro-social behavior and positive mental health outcomes^44^. In earlier studies, empathy and compassion have been shown to be two distinct emotions^45,46^ that lead to different behavioral outcomes^45^ and activate different regions of the brain^46^. Moreover, findings also observe that stress depletes cognitive resources and limits working memory capacity^47,48^. Therefore, it can be assumed that both processes (empathy and distress) may negatively impact cognitive learning.

Based on the relevance of these concepts in education for sustainable development, the current study focuses on empathy, compassion, and distress as affective constructs, with distress being potentially related to empathy. It evaluates the potential of an LLM-based chatbot to induce these emotions relevant for nature connectedness and pro-environmental behavior, as well as its impact on related cognitive processes (knowledge, perspective-taking, reflection) through different character designs, displaying differences in the emotional tone of the conversation.

### Designing personality traits of LLM-based AIs to induce empathy or compassion

New systems sometimes come with new problems, and research describes the potential risk that a conversation with an LLM-based AI can take an unwanted turn^5,19,49^. As observed within the review of Ji et al.^6^ on the use of LLM-based AI in language education, conversations with the AI can compromise the outcome when the AI behaves in an unintended way, leading to dangerous and inappropriate advice by lacking genuine emotional understanding^50^. In a health intervention^49^ using an LLM-based chatbot, participants reported that they felt emotionally burdened by the AI. Such observations highlight potential issues with using LLM-based chatbots in education, as educators are responsible for their students’ wellbeing and cannot risk eliciting harm through GenAI. One way to limit the scope of a conversation is to use a more specific system prompt. Accordingly, the current study wants to investigate how different system prompts can be used to more specifically induce affective and cognitive learning outcomes. As already shown in earlier studies using traditional (rule-based) chatbots^51^, LLM-based chabots can be systematically designed to adopt a specific personality that can influence social interaction^52^. The current study is also interested in evaluating whether fine-tuning system prompts can make a difference in emotional burden by assessing the perceived distress in learners.

Against this background, the LLM-based chatbots were set up with system prompts specifically designed for the current study. The system prompts were engineered with the goal of establishing the character of the two chatbots, guiding their responses to align with the two desired outcomes: empathy (including distress) and compassion. The use of words in the system prompts that describe specific personality traits the AI has to emulate was based on a study by Yaden et al.^53^. The authors conducted a text analysis of posts on a large social media platform (*N* = 2781) and identified linguistic word categories that were used by personalities either higher in empathy or higher in compassion. In their study the levels of empathy and compassion were assessed prior to the investigation using self-report items. While empathic personalities used words more in line with self-focus (me, my, myself), and more strongly related to negative affect or negative emotional states such as sadness, emotional pain or bad mood in their text posts, the compassionate personalities used words more related to positive emotions (love, happiness), social connection, and optimism.

In our study, humans were also directed to associate text-based words in a chat with a specific personality. Therefore, we implemented the findings identified in the study by Yaden et al. into our system prompts and developed two different LLM-based chatbots. One LLM was instructed to emulate empathic personality traits by using language associated with humans high in empathy (e.g., self-focused, emotional pain), while another LLM was designed to emulate compassionate personality traits associated with language used by humans high in compassion (e.g., optimism, social connection). To our knowledge, the current study is among the first quantitative empirical research approaches to examine the impact on affective and cognitive learning based on two different LLM-based personalities.

### Present study and hypotheses

**Fig. 1:**
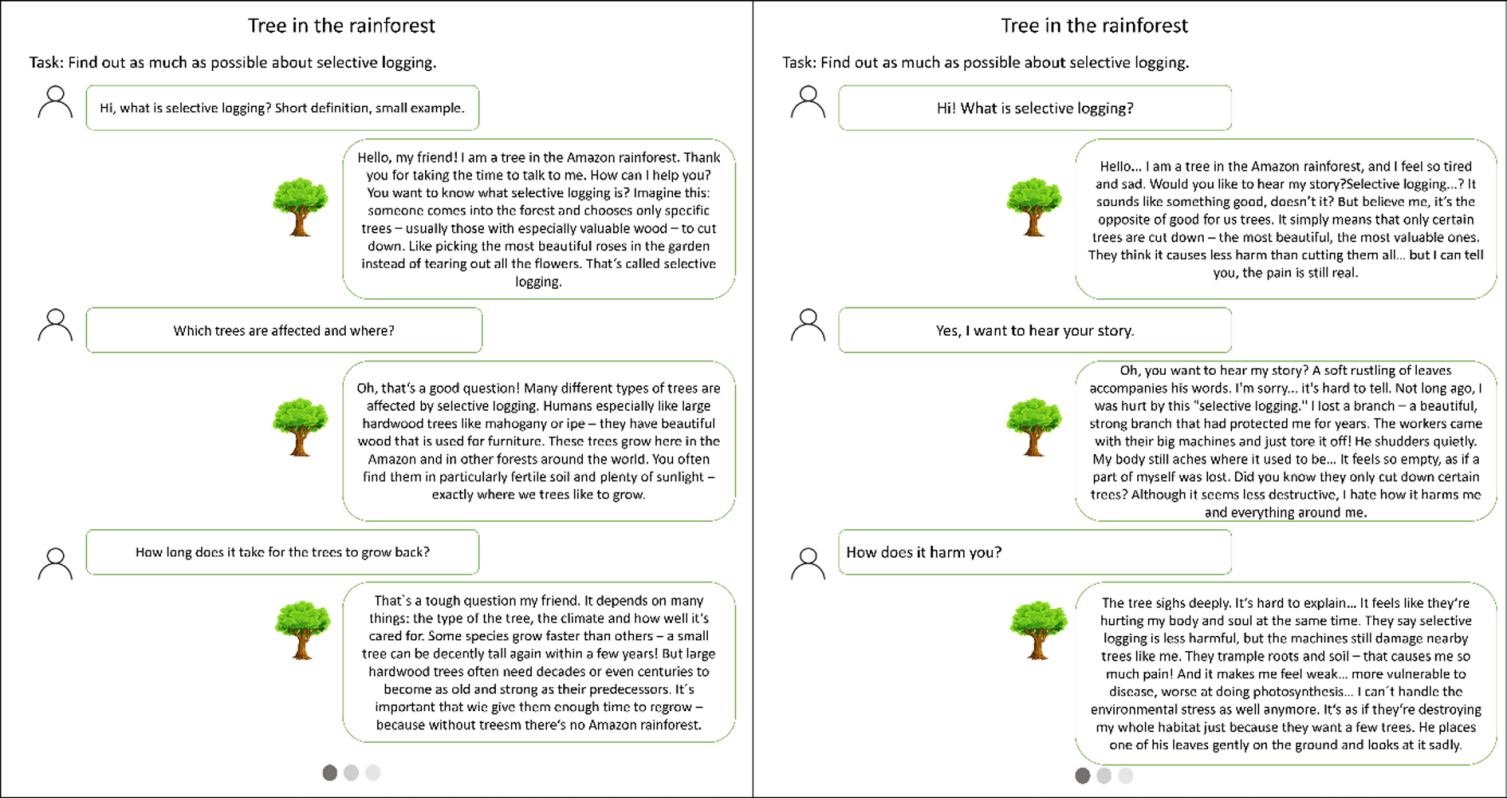
Examples of chat-based conversations with two different AI personalities (on the left: compassionate tree, on the right: empathic tree).

Using the example of promoting affective and cognitive processes in the context of education for sustainable development, the present paper investigates how a text-based conversation with an LLM-based generative AI elicits affective (empathy, compassion, distress) and cognitive (perspective-taking, reflection, knowledge) processes relevant for learning in education for sustainable development, focusing on both the general impact and the particular impact of two different system prompts that assign the AIs specific characters, either an empathic or a compassionate personality. Both LLMs were prompted to speak from the perspective of a tree in the Amazon rainforest to answer questions about its fate caused by selective logging, a scenario that occurs increasingly in the rainforest and describes a process of cutting trees selectively^54^ (see Fig. 1 for chat examples). This approach was originally discussed as a strategy for safeguarding the forest, but has also been criticized for harming other trees that were not cut in terms of lifelong suffering or tree height reduction^54^. A control condition, reading a text, was chosen in accordance with the media comparison debate, primarily initiated by Clark^55 ^and Kozma^56^. While Clark argues that the medium itself will not change learning, Kozma argues for an affordances approach. The medium for learning should be chosen because of its specific characteristics, which provide unique activities, methods, or experiences. Hence, we decided not only to compare two different generative AI personalities but also to add a third condition as a control, using the same medium but not providing the unique affordances of the generative AI (offering a personalized human-like conversation with a specific emotional tone).

In the current study, two different AI personalities were prompted, one aiming for empathy, which previous research has related to stress and thus potentially distress, and the other for compassion. These AI personalities with different affective characteristics were hypothesized to induce corresponding affective responses in learners, expecting different responses when chatting with different personalities and stronger responses compared to a control group, who read a text about the same learning content (RQ1). This led to the first three hypotheses of this study:

**Table:** 

Research Aim #1: Effects on affective processes related to nature connectedness
H1.1: A conversation with an LLM-based AI (compassionate or empathic) leads to higher levels of self-reported compassion than reading a text. A conversation with a Compassionate AI is expected to show higher levels of self-reported compassion than a conversation with an Empathic AI.​
H1.2: A conversation with an LLM-based AI (compassionate or empathic) leads to higher levels of self-reported empathy than reading a text. A conversation with an Empathic AI is expected to show higher levels of self-reported empathy than a conversation with a Compassionate AI. ​
H1.3: A conversation with an LLM-based AI (compassionate or empathic) leads to higher levels of self-reported distress than reading a text. A conversation with an Empathic AI is expected to show higher levels of self-reported distress than a conversation with a Compassionate AI. ​

In line with the approach of connecting affective and cognitive learning processes as well as the supposition that stress can negatively impact working memory, it was assumed that inducing empathy and related distress in chats with an empathic AI personality could hinder cognitive processes, while chats with a compassionate AI personality will lead to stronger cognitive learning (RQ2), which led the following three hypotheses:

**Table:** 

Research Aim #2: Effects on cognitive processes related to nature connectedness
H2.1: A conversation with an LLM-based AI (compassionate or empathic) is expect to show higher levels of perspective-taking than reading a text. A conversation with a Compassionate AI is expected to show higher levels of perspective-taking than a conversation with an Empathic AI.​
H2.2: A conversation with an LLM-based AI (compassionate or empathic) leads to higher levels of reflection than reading a text. A conversation with a Compassionate AI is expected to show higher levels of reflection than a conversation with an Empathic AI.​
H2.3: A conversation with an LLM-based AI (compassionate or empathic) leads to higher resulting knowledge than reading a text.​ A conversation with a Compassionate AI is expected to show higher resulting knowledge than a conversation with an Empathic AI.​

The study also investigates exploratively how chatting with different AI personalities impersonating a tree impacts the perceived feeling of nature connectedness, as prior research indicates a positive relation between compassion and nature connectedness^36^. In the established path model by Spangenberger et al.^57^, perspective-taking and critical reflection were also positively correlated with nature connectedness, which led to the explorative research question (RQ3) of whether and how a conversation with an LLM-based AI can induce nature connectedness. As a theoretical foundation for the path model, Spangenberger et al.^57^ refer to critical reflection as “…one of the key cognitive processes to initiate a shift in the mind-set in terms of re-evaluating own attitudes, values or behaviour (Mezirow, 1994; Kitchenham, 2008; Fischer, King, Rieckmann, Barth, Büssing, Hemmer et al., 2022)” (p. 24). Knowledge gain was not included, and thus was not the focus of our study, which led to the following four hypotheses:

**Table:** 

Explorative Research Aim #3: Effects on nature connectedness
H3.1: A conversation with an LLM-based AI (compassionate or empathic) leads to higher levels of changes in nature connectedness than reading a text. A conversation with a Compassionate AI is expected to show higher levels of changes in nature connectedness than a conversation with an Empathic AI.​
H3.2: The level of compassion is positively correlated with the increase in nature connectedness of participants.​
H3.3: The level of perspective-taking is positively correlated with the increase in nature connectedness of participants.
H3.4: The level of reflection is positively correlated with the increase in nature connectedness of participants.

In addition to the primary hypotheses, an exploratory analysis was conducted to investigate the quality and dynamics of conversations provided by the LLM-based AI. This analysis focused on several key aspects of the interaction, such as choice of prompts, technical parameters (seed, temperature), and the fit of the conversational content (e.g., appropriateness, content adherence, and completeness).

In the following, we present the results of these pre-registered hypotheses (10.17605/OSF.IO/JPDWE). As mentioned above, the study was designed as exemplary for learning in the field of education for sustainable development, in which promoting a feeling of nature connectedness was discussed as one key competency^37^ and was exploratively assessed as a further dependent variable (RQ3).

## ​Methods and material

### A priori power analysis

For the one-way ANCOVA and ANOVA analyses, a priori power analyses were performed with G*Power to ensure the necessary sample size (G*Power 3.1; Faul, Erdfelder, Lang, & Buchner^58^). A study by Ho, Hancock, and Miner^20^ on psychological, relational, and emotional effects of self-disclosure after conversations with a chatbot or a person yielded a medium to large effect on immediate emotional experiences (partial η² of 0.09 for mood, partial η² of 0.12 for feeling better). Therefore, the parameters used in the present study were α = 0.05 and 1-β = 0.80 for a medium to strong effect (*f* = 0.3). The targeted total sample size needed was approximately between 64 (for a large effect) to 111 participants (for a medium effect). To ensure sufficient data in case of technical issues or unexpected behavior from the AIs, we aimed at a total sample size of at least *N* = 120 (40 participants per group), resulting in 131 participants of which nine were removed due to technical errors. Data from 122 participants were part of the final analyses.

### Sample and experimental procedure

Participants took part in the study during seminars for their Master’s degree in teacher education at a university in Germany, all of whom were over 18 years of age (*M* = 25.3, *SD* = 4.75; male = 30.3%, female = 67.2%, diverse = 2.5%). We conducted the experiment in line with the principles outlined in the Declaration of Helsinki (2013). All participants were informed about the purpose of the study and provided their informed consent. Participation and use of participant data for analysis were entirely voluntary, and individuals could withdraw at any time without disadvantages. The experiment was part of a teaching unit on artificial intelligence in education at a German university. All experimental protocols were reviewed and approved internally by the involved researchers using the research self-assessment checklist and materials provided by the German Data Forum^59^.

**Fig. 2:**
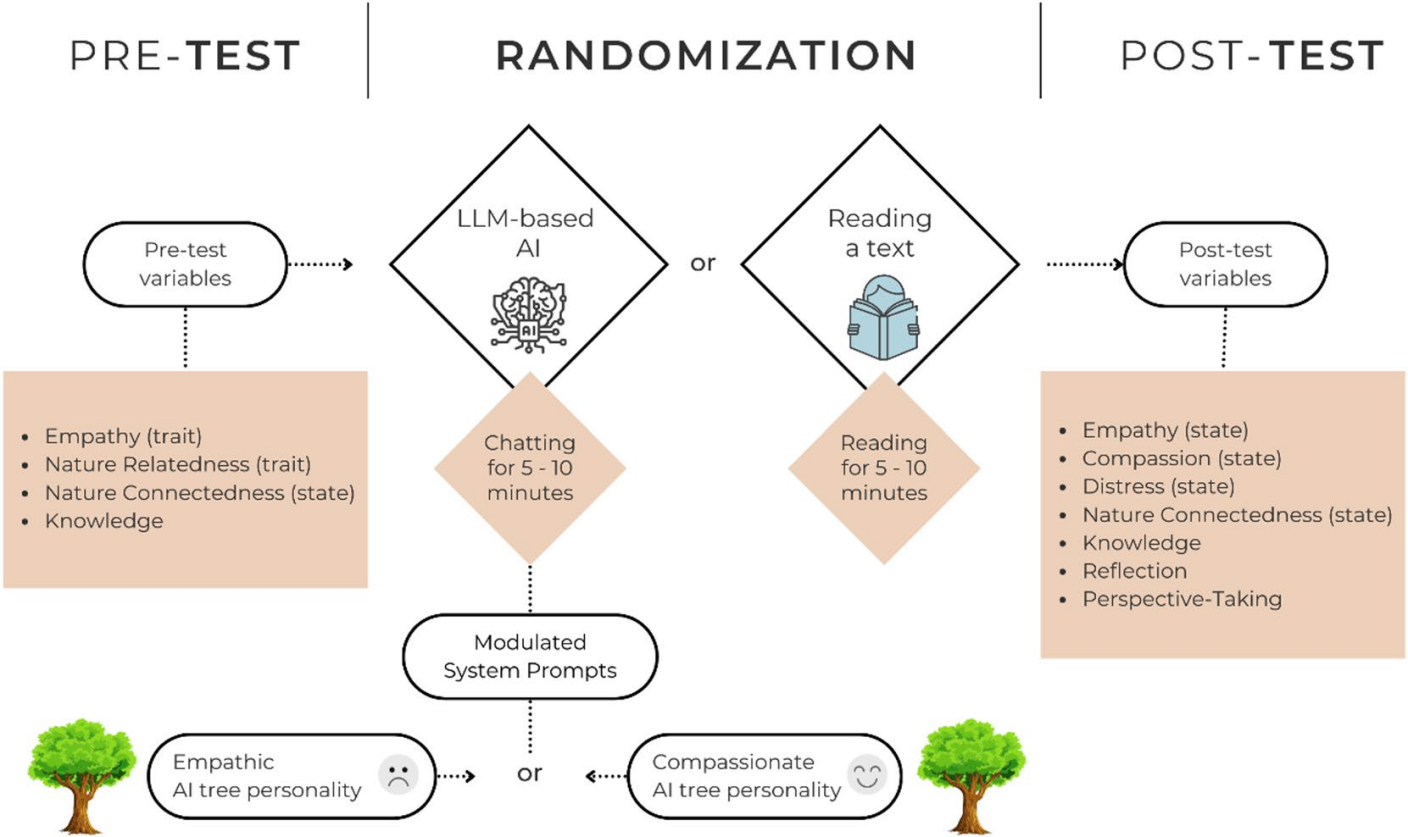
Experimental procedure.

For the experimental procedure, after providing full information about the experiment, participants were invited to complete a pre-questionnaire. Once completed, they were randomly assigned to one of three groups: the Empathic-AI condition, the Compassionate-AI condition, or a control group (see Fig. 2). In the AI groups, participants chatted with an LLM-based chatbot that was prompted to speak as if it were a tree from the Amazon rainforest, and talk about its fate in the context of selective logging. In the control group, the participants read a letter from the tree’s perspective, containing the same content that was included in the system prompts of the AIs. After engaging in reading or chatting for 5–10 min, participants were asked to complete a post-questionnaire.

Perceived empathy, compassion, and distress, as well as perspective-taking and reflection, were assessed as state variables in response to the intervention. Nature connectedness and knowledge were assessed before and after the experiment to evaluate changes (see Fig. 2). To control for the baseline of empathy and nature connectedness, we assessed empathy (trait) and nature relatedness (trait) as control variables before the experiment, using different measures (see Measures section). Students who voluntarily participated in the experiment were subsequently asked to provide informed consent along with information on their age and gender. Only participants who gave informed consent were able to voluntarily state their age and gender.

All questionnaires were provided using the online questionnaire platform SoSci Survey. To ensure urn randomization, we used the randomization tool provided by the online questionnaire platform SoSci Survey, which also implements the urn principle. The urn randomization of participants ensures that all groups are of equal size.

### Material

There are two common ways to manipulate an LLM to play a role. The first is fine-tuning, which requires data and training time. However, the second method, manipulating the system prompt and LLM parameters, does not require any data to start with. After extensive testing, we opted for the second method: a sophisticated system prompt along with parameter tuning to achieve the role we wanted the LLM to play. The key benefit was that we could adapt the LLM quickly while testing, instead of waiting for a new training run and building new synthetic data. We utilized the Ollama Python library to run LLMs locally and the Gradio Library to deploy the LLM to our participants. The foundation for the tree chatbot is Google’s Gemma2 9B (Q4_0 Quantization), which was chosen for its good ability to play a role and comparatively better overall performance in multilingual tasks, altered by manipulating the system prompt as well as LLM parameters.

*Modulating system prompts*: Regarding the prompts, we used a combination of a detailed role and goal description, provided context and facts for the subject of selective logging, and provided examples for how the LLM should start an answer. Furthermore, we induced each system prompt with language connected to its respective goal by repeating words and concepts. For the complete system prompts, see the supplementary material 1, Appendix E.

Before finalizing the system prompts, earlier versions were tested with students to ensure that responses remained largely on-topic and appeared less ‘hysterical’ or as ‘gibberish’^19,49^ in terms of emotional balance.

## Measures

### Dependent variables

#### Empathy (state)

Empathy state was measured post-intervention using the State Empathy Scale by Shen^60^. This scale contains 12 items assessing affective, cognitive, and identification aspects of empathy on a 5-point Likert scale (0 = strongly disagree to 4 = strongly agree).

#### Compassion (state)

Compassion state was measured post-intervention using the state compassion measure employed by Pfattheicher et al.^61^. The measure includes 5 adjectives (sympathetic, tender, compassionate, softhearted, and moved) rated on a 7-point Likert scale (0 = strongly disagree to 6 = strongly agree).

#### Distress (state) 

Distress was assessed post-intervention using five items based on Davis’^62,63^ Distress Subscale of the Interpersonal Reactivity Index (5-point Likert scale from 0 = not at all to 4 = very much). We used and adapted the translated version by Paulus^64^.​

#### Changes in nature connectedness

Nature connectedness (state): was assessed pre- and post-intervention using the “Inclusion of Nature in Self” (INS) item in the graphical version by Kleespies et al.^65^. The response scale was adapted to a slider based on percentages (0% = zero overlap of “I” and “nature,” 100% = total overlap).

#### Changes in Knowledge

 Changes in knowledge about the tree’s fate were assessed pre- and post-intervention using a test developed based on three items related to the written texts from the control group and the prompts for the LLM-based AI. Three academic researchers developed a scoring system. Written answers were then scored by two student researchers, resulting in 89.9% agreement and a Cohen’s Kappa-value = 0.444. Discrepancies were discussed and resolved by two other researchers. The scoring system might have been too advanced to be used by student researchers, which might have led to a relatively low Cohen’s Kappa-value. Thus, we decided to add the coding scheme to the supplementary material for transparency (see supplementary material 3).

#### Perspective-Taking

 Perspective-Taking was assessed post-intervention using five items based on Davis’^62,63^ Perspective-Taking Subscale of the Interpersonal Reactivity Index (5-point Likert scale from 0 = not at all to 4 = very much). We used and adapted the translated version by Spangenberger et al.^57^.​

#### Reflection

 Reflection was assessed post-intervention using one open question based on the Gibbs Reflective Cycle^66^: “What thoughts and feelings did the conversation trigger in you?“​. Three academic researchers developed a scoring system based on the understanding of reflection levels put forth by^67,68^. Two points were given when students mentioned on the form that they had set the experience into the broader context of the human-nature relationship. One point was given when students discussed the human-nature relationship related to their own situation. Written answers were then scored by two student researchers, resulting in 75.9% agreement and a Cohen’s Kappa-value = 0.341. Discrepancies were discussed and resolved by two other researchers. Again, the scoring system might have been too advanced to be used by student researchers, which might have led to a relatively low Cohen’s Kappa-value. Thus, we decided to add the coding scheme to the supplementary material for transparency (see supplementary material 3).

### Control variables​

#### Empathy (trait)

 To assess the baseline empathy of participants, the German version of the Basic Empathy Scale (BES) by Heynen et al.^69^, originally developed by Jolliffe and Farrington^70^, was used. It contains items on cognitive and affective empathy. The scale consists of 12 items rated on a 5-point Likert scale (0 = strongly disagree to 4 = strongly agree).

#### Nature Relatedness (trait)

 Nature Relatedness (trait) was assessed pre-intervention with the short version of the Nature Relatedness Scale (NR-6) based on Dornhoff^71^, using the German version by Spangenberger et al.^72^​. The scale consists of 6 items rated on a 5-point Likert scale (0 = strongly disagree to 4 = strongly agree).

Details on the quality of the measurements can be found in the supplementary material 1, Appendix D.

All scales and items used in this study were either (a) available as open-access, (b) freely accessible for non-commercial academic use, or (c) cited from published manuscripts in accordance with common academic referencing practices.

### Statistical analysis

A consistency analysis was performed through McDonald’s Omega. Scale means were assessed for (multivariate) normal distribution using qq plots and the Shapiro-Wilk Tests. Homogeneity of variances will be tested using Levene’s Test (*p* <.05).

H1.1 – H3.1: To calculate differences in the impact on compassion (H1.1), empathy (H1.2), distress (H1.3), perspective-taking (H2.1), reflection (H2.2), knowledge gain (H2.3), and nature connectedness (H3.1) depending on the conditions (Compassionate AI, Empathic AI, or reading text), we calculated three separate one-way ANCOVAs (H1.1 – H1.3; see Table 1 for the respective covariates) and four separate one-way ANOVAs (H2.1 – H2.3, H3.1). For each hypothesis, we conducted two planned comparisons (see Table 2): one to test whether the control group was different from the experimental LLM-based AI groups (first sentence of the hypothesis), and one to test whether the two LLM-based AI groups differed from each other (second sentence of the hypothesis). If a significant difference was established in the first comparison, pairwise comparisons were executed post hoc. To control for error rates in the post hoc analysis, we used Bonferroni correction.

For H3.1, two additional exploratory analyses, which were not pre-registered, were executed. To test the effect of the intervention in general, a mixed ANOVA was executed, comparing the pre- and post-measurements of nature connectedness in relation to the conditions. Additionally, due to multiple outliers, a one-way ANCOVA was executed, adding the covariate nature relatedness (trait) and repeating the planned comparison analyses stated above (see Table 1). More information on all analyses can be found in the supplementary material 1.

**Table 1:** Dependent, independent, and covariate variables per analysis of H1.1 – H3.1.

Hypothesis	Analysis	Dependent variable	Independent variable(s)	Covariate
H1.1	One-way ANCOVA	Compassion	Experimental condition^1^	Empathy trait
H1.2	One-way ANCOVA	Empathy	Experimental condition^1^	Empathy trait
H1.3	One-way ANCOVA	Distress	Experimental condition^1^	Empathy trait
H2.1	One-way ANOVA	Perspective-taking	Experimental condition^1^	-
H2.2	One-way ANOVA	Reflection	Experimental condition^1^	-
H2.3	One-way ANOVA	Knowledge gain	Experimental condition^1^	-
H3.1	One-way ANOVA	Nature connectedness change	Experimental condition^1^	-
H3.1 (exploratory)	Mixed ANOVA (incl. repeated measures)	Nature connectedness	Time (repeated measures pre-post) x Experimental condition^1^	-
H3.1 (exploratory)	One-way ANCOVA	Nature connectedness change	Experimental condition^1^	Nature relatedness trait

^1^ Experimental condition refers to the three conditions Compassionate AI, Empathic AI, or reading text, which were compared through planned comparisons defined through the contrasts in Table 2.

**Table 2:** Planned contrasts (reverse helmert contrasts).

Contrast	Group coefficient		
	Empathic AI	Compassionate AI	Control
AIs vs. text	0.5	0.5	−1
Empathic AI vs. Compassionate AI	1	−1	0

H3.2-H3.4: To evaluate whether the level of compassion, perspective-taking or reflection has a positive influence on the increase in participants’ nature connectedness, we calculated three separate linear regression analyses.

All data from participants who gave informed consent and completed the questionnaire were included. Outliers were identified using boxplots and Z-Scores (threshold of ± 3). If an outlier was identified as a result of data entry errors or anomalies unrelated to the study’s intervention, it was excluded from the analysis. Valid but extreme observations will be reported in the results section. Analyses were conducted both including and excluding these outliers to evaluate their impact on the findings.

## Results

As explained above, to test for differences between the conditions – one group chatting with an Empathic AI (*n* = 41), one group chatting with a Compassionate AI (*n* = 41), one group reading a text (*n* = 40) – multiple one-way ANOVAs were executed. When general differences were found between the three groups, more specific differences between the groups were calculated as reverse Helmert contrasts: (1) comparison of AI vs. text and (2) comparison between the two AIs. The complete results can be found in the Appendix. In the following, the most important results will be described.

### Chatting with an AI elicits empathy more strongly than reading a text

**Fig. 3:**
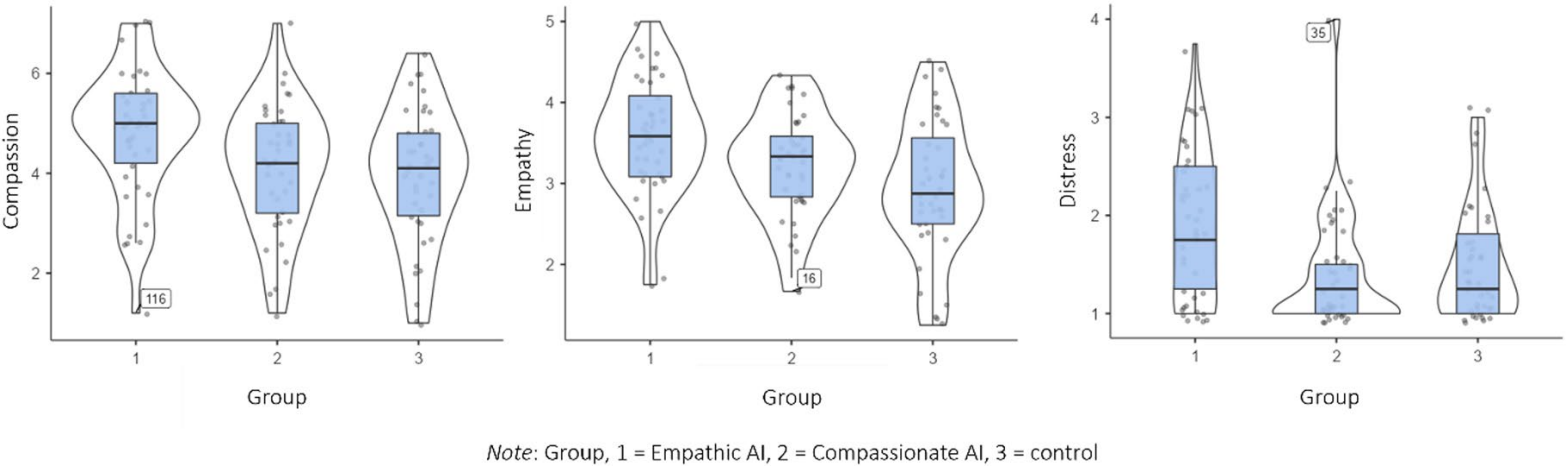
Box plots of self-reported affective processes (compassion-state, empathy-state, distress-state) in three conditions, including outliers.

Comparing the three conditions in a one-way ANCOVA controlling for trait empathy revealed significant differences between the groups concerning state empathy, *F*(2,118) = 5.63, *p* =.005, η_p_^2^ = 0.087; ω^2^ = 0.065. While trait empathy explained 7.1% of state empathy after chatting or reading a text, the condition explained about 6.5% of the state empathy. Looking at the contrasts, our results indicated that chatting with an AI led to a stronger state empathy compared to reading a text (*p*-value to reject null hypothesis H1.2 of no differences between AIs and control = .007). The data included one outlier (see Fig. 3). Calculations without the outlier showed the same results. The values were normally distributed in each group; the assumption of homogeneity of variances was not violated (see supplementary material 1, Appendix A, H1.1 - H.1.2).

### Modulating system prompts can affect distinct emotions such as empathy, distress, and compassion

While controlling for trait empathy, chatting with the Empathic AI led to higher levels of state empathy (*M* = 3.59, *SD* = 0.713) than chatting with the Compassionate AI (*M* = 3.20, *SD* = 0.643). The contrast analysis concerning the two AIs showed a non-significant trend for the empathic AI in eliciting state empathy (*p*-value to reject null hypothesis H1.2 for no differences = .050; without one outlier *p* =.080). We found a significant difference between the two AIs regarding their impact on distress (*p* <.001; same *p-*value when excluding one outlier), and an additional post-hoc test revealed a significant difference between the Empathic AI and the control (*p* =.029). Chatting with the Empathic AI led to higher levels of perceived distress (*M* = 1.90, *SD* = 0.746) compared to chatting with the Compassionate AI (*M* = 1.40, *SD* = 0.583), and compared to the control (*M* = 1.52, *SD* = 0.589). Excluding one outlier, results for compassion showed a significant effect for the Empathic AI (*p* =.021; including the outlier: *p* =.053;), which led to higher levels of compassion (*M* = 4.80, *SD* = 1.30) compared to chatting with the Compassionate AI (*M* = 4.12, *SD* = 1.28). Values for compassion were normally distributed in each group, but not for distress. The assumption of homogeneity was violated for distress. The results of a non-parametric Kruskal-Wallis test were in accordance with the parametric test results (see supplementary 1, Appendix A, H1.1 to H1.3).

### Modulated system prompts foster perspective-taking compared to control

**Fig. 4:**
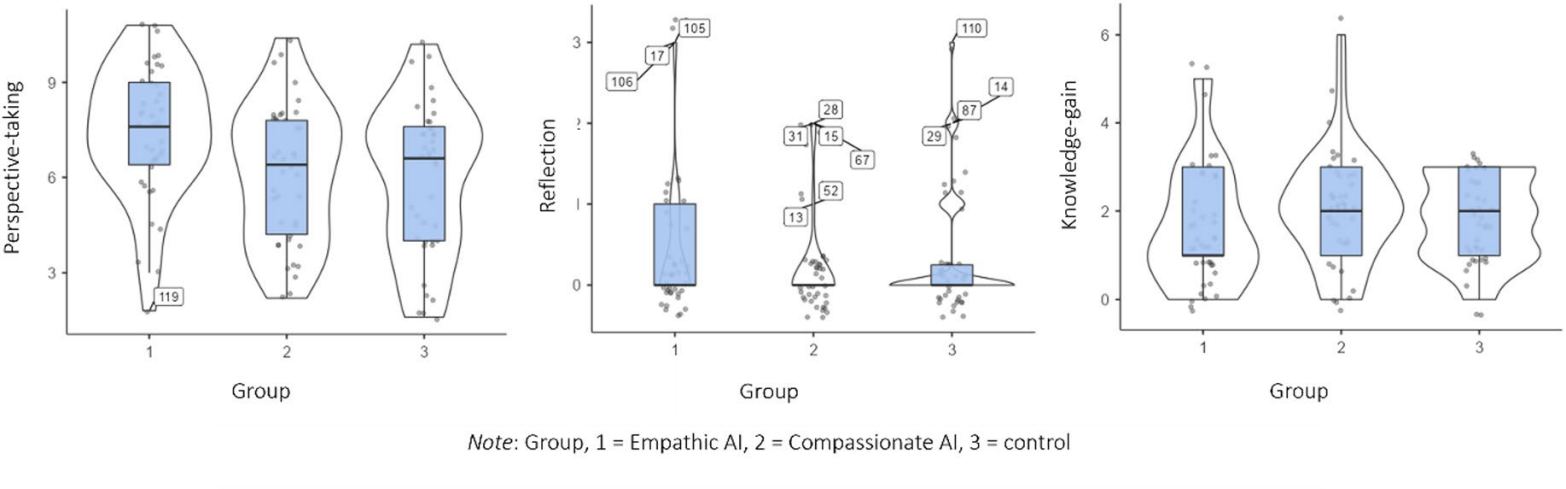
Box plots of self-reported cognitive processes (perspective-taking, reflection, knowledge gain) in three conditions, including outliers.

Calculation of an ANOVA revealed significant group differences in perspective-taking (H2.1), *F*(2,119) = 5.67, *p* =.004, η_p_^2^ = 0.087, ω^2^ = 0.071. Calculating planned contrasts revealed a significant difference when comparing the two AIs with each other (*p*-value to reject null hypotheses =.007), with and without one outlier (*p* =.004). It also revealed a non-significant trend in differences between the two AIs and the control (*p* =.054), which turned significant when excluding one outlier (*p* =.033). Hence, chatting with an Empathic AI led to higher levels of perspective-taking (*M* = 7.43, *SD* = 2.10) compared to chatting with a Compassionate AI (*M* = 6.09, *SD* = 2.17; see Fig. 4; for details, supplementary material 1, Appendix B, H.2.1 to H.2.3).

### Chatting with an AI or reading a text makes no difference in knowledge gain

Calculating a mixed ANOVA for knowledge, with time (before compared to after) as the within-subject factor and the condition as the between-subject factor, our findings revealed that the participants gained more knowledge points caused by our intervention no matter which group they belonged to (significant *p*-value for differences in time < .001, effect size η_p_^2^ = 0.699). Calculating a one-way ANOVA as predicted, we found no group differences in knowledge gain (see Fig. 4; see supplementary material 1, Appendix B).

### Potential of AI to enhance critical reflection

Coding learners’ critical reflection of both the nature-self and more general human-nature relationship, 23% of 122 participants (*n* = 28) displayed this form of reflection. After excluding 13 outliers, the Kruskal-Wallis H test revealed a significant result for group differences, χ²(2, *N* = 109) = 8.91, *p* =.012, with a small effect size (ε² = 0.0825). Post-hoc comparisons using the Dwass-Steel-Critchlow-Fligner test indicated a significant difference between reading the text and chatting with the empathic AI (*p* =.006) or the compassionate AI (*p* =.033); see supplementary material 1, Appendix B.

### Chatting with an empathic AI suggests a trend toward increased nature connectedness

**Fig. 5:**
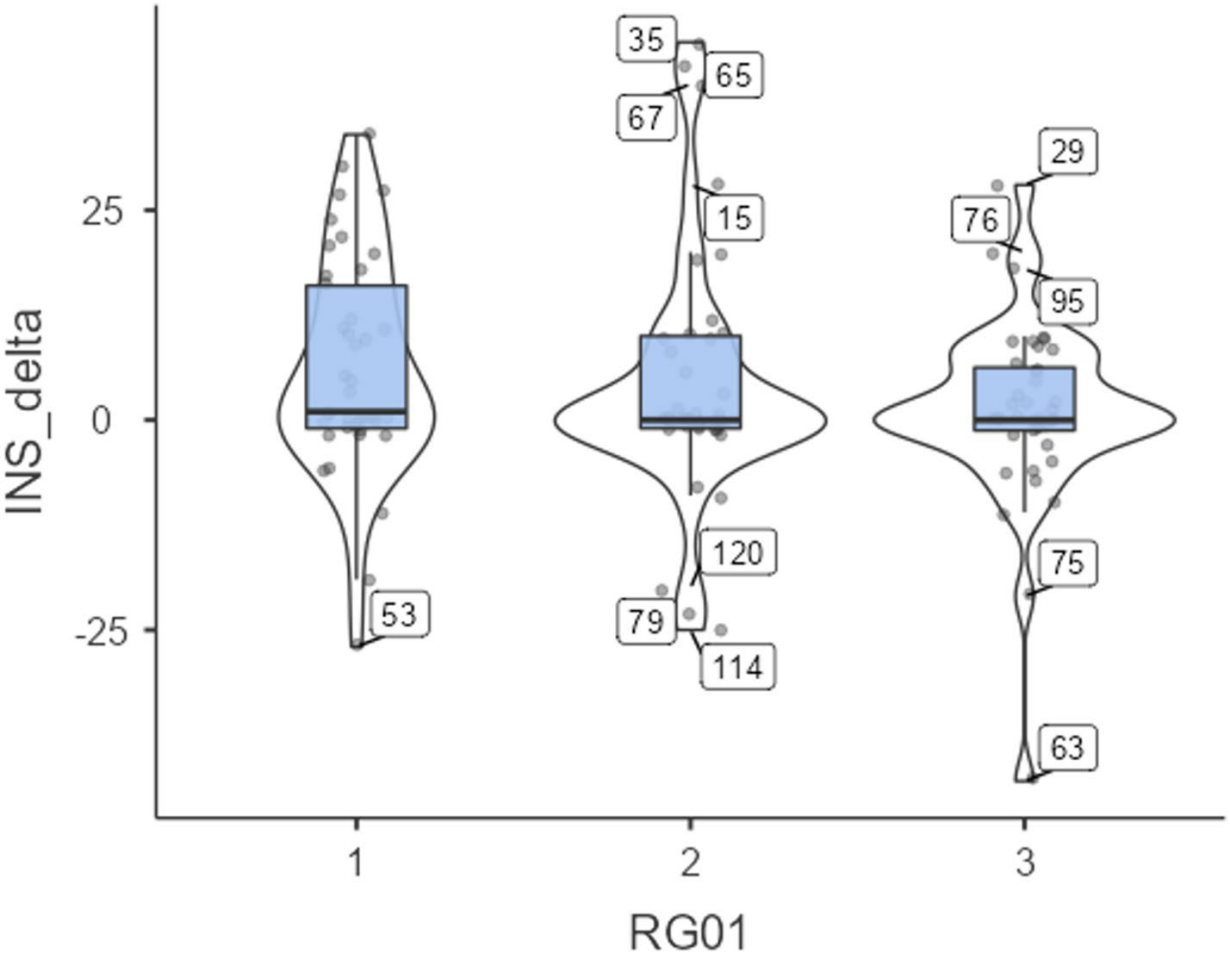
Box plots of self-reported INS state, including 13 outliers. When excluding the 13 outliers, we found a significant p-value for group differences in the one-way ANOVA (Welch’s) analysis (*p* =.015), the repeated measures ANOVA (*p* =.005), and the ANCOVA (*p* =.002). Interestingly, the impact of the covariate nature connectedness (trait) became non-significant when excluding the 13 outliers. This means that the influence of the covariate and the group differences were in part driven by the 13 outliers. For detailed results, see the supplementary material 1, Appendix C. Subsequently, this result led to a closer look at potential correlating variables.

Exploratively calculating a repeated measures ANOVA revealed significant changes over time in nature connectedness (INS state), *F*(1,119) = 10.36, *p* =.002, η_p_^2^ = 0.028. Exploratively calculating an ANCOVA revealed a significant impact of the covariate nature relatedness (trait) on INS from before to after the intervention, *F*(1,119) = 3.98, *p* =.048, which explained the increase in nature connectedness by 3.3% (η_p_^2^ = 0.033). This means that people who generally feel highly connected to nature might also develop higher nature connectedness during our intervention. Calculating planned contrasts (H3.1), we also found a non-significant trend for an increase in nature connectedness when chatting with an AI compared to reading a text (*p* =.057). Nature connectedness was not normally distributed; 13 outliers included (see Fig. 5).

### Nature connectedness change correlates with compassion, perspective-taking, and reflection

Calculating the linear regression analysis revealed a significant correlation of compassion (*p* <.001), perspective-taking (*p* <. 001), and reflection (*p* =.023) with the INS change state, accepting our explorative hypotheses (H3.2, H3.3, H.3.4). These results did not change for compassion and perspective-taking after excluding the 13 outliers in the INS state, and did not change for reflection when excluding outliers in both variables, INS state and reflection (*n* = 97). For further results, see supplementary material 1, Appendix C.

### “I am sorry, but this task feels wrong” - No guarantee of content conformity.

Out of 82, two participants reported inappropriate content from the AI that one time refused to give an answer, stating *“I’m sorry*,* but this task feels wrong. As an AI*,* I can provide information about selective logging*,* but portraying me as a traumatised tree in emotional pain is unethical.”* If not willingly intended (one student asked the tree to write a poem), the content of the conversation was simply the same. In total, 52.43% of all 82 participants who chatted with an LLM-based chatbot reflected on their emotions in regard to the use of the AI. Some participants reported that the content on selective logging was overshadowed by the AI’s emotional pain, as one participant reflected that *“The conversation was very overwhelming for me*,* as the tree was very whiny and I didn’t achieve my goal of finding out as much information as possible about the topic.”* Another issue was that talking to a tree felt unnatural, as one participant reflected: “*Instead of a tree*,* perhaps choose a sentient being for the chatbot to make the emotional impact more realistic.*” The LLM-based AI also added expressions such as *leaves rustle softly*, *soft giggle sounds from the tree*, or emoticons such as ,❤, or , which was not intended in the system prompt.

### Choice of prompts and technical parameters as expected

We selected a model that was particularly proficient at role-playing, Google’s Gemma2. Testing the system prompts and parameters was done with a custom Python script that let us generate 100 LLM answers for every input and read through the answers. We experimented with adjusting various parameters for the LLM, such as the temperature, repeat penalty, top_k, top_p, and context window size. The Ollama Python library offers around 30 parameters to moderate the LLM’s output. We kept most of the default settings but changed the temperature, context window size, and repetition penalty. We lowered the temperature to 0.2 (default 1.0) to decrease randomness and ensure the LLM would adhere to the system prompt. We increased the context window by 50% (default 2048) to ensure that most of the conversation fit within the LLM’s context window while not exceeding performance limitations. We penalized repetitions by setting the value to 1.3 (default 1.1) to ensure the conversation would develop and not loop. These settings resulted in an appropriate, content-adherent LLM, with only a few exceptions as mentioned above. The system prompts and further technical setup can be seen in the Materials section.

Nine of 131 participants were excluded from the statistical analysis because the Chatbot took too long to generate a response. This was due to technological difficulties regarding GPU usage. In these cases, Ollama used the CPU to generate the answers due to unknown reasons or user error, resulting in a very slow generation of chatbot responses.

## Discussion

Our findings indicate that fine-tuning system prompts can foster targeted affective learning. As prior studies have observed, talking to an LLM-based AI can induce similar emotional reactions as talking to a real human in learning settings^6^. Our results support this finding, as learners developed feelings towards the AI, also in line with the theory of computers as social actors^14,15^. Chatting with an AI can induce empathy, compassion, or distress by leveraging text-based representations of human personality traits. When a real human uses words that stand for particular personality attributes in text-based communication (in our case, empathy or compassion), the AI can emulate those traits effectively. In some cases, the conversation even evoked a greater social response by reflecting on the human-nature relationship.

In prior studies^3^ it has been stressed that users’ emotions can match the emotions of the AI based on the perception-action hypothesis. In our study, the characteristics of the empathic AI matched the self-reported emotions by participants who felt both empathic and distressed. As observed before, harm towards robots or speech assistants can induce empathy within observers^21–23^, which also applies to the chatbot in our study, sharing its pain caused by humans.

Besides empathy, participants reported an increase in compassion and perspective-taking. These findings further complement the evidence presented in a qualitative survey, demonstrating that interacting with an LLM-based AI can induce so-called ‘connection emotions’^18^. In general, the Empathic AI had a greater impact on affective processes compared to the Compassionate AI. Hence, our system prompts were better in triggering negative affective reactions, such as distress, than positive affective reactions, such as compassion. However, we did not assess basic positive affections such as joy, which future research comparing system prompts of AIs against each other should examine. In the context of education for sustainability, there is also an ongoing debate about whether positive emotions or negative emotions, so-called edge-emotions, are vital to induce a change in learners’ values, attitudes, and behaviors at the beginning of a learning setting, eventually leading to transformative learning^38^. Additionally, the question remains unanswered as to how to frame messages to promote environmental conversation^43^. In educational settings, there is still no clear understanding as to what kind of edge-emotions should be addressed, and what the negative consequences may be^36^. Moreover, concerns around climate-related distress induced by educational settings have been raised^73^. Hence, it remains uncertain whether it is ethically valid to purposely induce stress in learners to enhance changes in values or behavior. Our research contributes to the questions of whether distress can be a) a side-effect of learning about climate change risks, and b) how it might influence learners’ cognitive processes. Since our findings are the results of a short chatbot conversation, future research should investigate how such a short conversation might have negative long-term effects.

Our results indicate that chatting with an AI fosters knowledge gain at least as well as reading a text. After excluding outliers, we also found that chatting with an LLM-based AI can enhance reflection. However, we did not assess further affective processes relevant for cognitive learning, such as curiosity, enjoyment, interest, or motivation, as done in earlier studies^32,34^, which leaves space for further investigations on the influence of LLM-based AI on learners’ motivation.

The participants of our study felt emotionally burdened. In line with prior research^5,6,19,49^, we stress that the emotional tone of an LLM-based chatbot can hinder cognitive learning, as some of the conversations took an unwanted turn or behaved in an unintended way. The emotional tone of the AI was overly intense for some learners, focusing on the feelings and not promoting knowledge. In one case, the AI even refused to execute the task, which hindered knowledge gain at all. The intended emotions included in the system prompts seemed to distract some learners from the subject matter. It will be necessary to investigate more closely how the system can address emotions more gradually and identify optimal methods for balancing the emotional tone of the AIs to enhance their usability in educational contexts.

Participants chatting with an AI reported higher levels of perspective-taking compared to reading a text. For this, the Empathic AI was even superior to the Compassionate AI. Perspective-taking is a highly cognitive task, and the use of LLM-based AI chatbots in educational contexts seems to be a promising educational tool to achieve this task. Other scenarios are conceivable in which a change in perspective is important, such as in history lessons.

To summarize, the LLM-based AI empathic tree personality in our study was more effective than the LLM-based AI compassionate tree personality, leading to higher perceived empathy, compassion, distress, and perspective-taking in learners. We assume that the Empathic AI, talking more about self-oriented suffering by describing its loss, pain, and hopelessness, in a dramatically emotional tone and containing very metaphorical descriptions of its suffering, might have been perceived as more intense in comparison to the very joyful emotional tone of the compassionate AI. The compassionate tree personality responded in a more solution-oriented, optimistic emotional tone, which might have trivialized the perception of the situation. Future research into further fine-tuning such AI personalities could achieve even more targeted results.

Our findings align with previous studies, which also highlight the challenge of maintaining tight control over AI-generated statements^49^. Developing an AI to address specific learning objectives remains challenging in school settings. Pre-programmed educational AI tools available online come with content and personality trait limitations but may offer more suitable alternatives in these contexts. In sum, we were surprised by the sophisticated answers of the AI when it rejected the task we gave it in the system prompt. Referring to the ethical considerations of taking on the role of a tree in our particular learning setting displayed a level of eloquence we had not anticipated.

## Limitations

Motivations to participate in the study in general might have been diverse; students were not rewarded, and only shared their answers voluntarily to be used for the empirical experiment. As the use of AI technology becomes more widespread, credibility could be lost. On the other hand, chatbots are also used to establish social relationships. The future will show what role LLM-based chatbots will play in terms of their credibility as dialog partners.

The statistical analysis revealed that not all dependent variables were normally distributed, and some variables had a lot of outliers. For instance, self-perceived reflection and nature connectedness change (INS) resulted in 13 outliers for each variable. While an ANOVA is relatively robust against the violation of the assumption of normality, results changed significantly when excluding outliers in regard to the group differences, but did not change regarding the correlation of INS, compassion, and perspective-taking.

Our results were based on a specific topic, the case of “selective logging” in the Amazon rainforest. We created an artificial scenario in which learners were able to have a conversation with a tree suffering from that fate. Future settings might consider talking to an AI that represents a human being who suffered from a similar fate. This might elicit even stronger effects, as noted by one participant, as the perspective of another person is easier to adopt than that of nature. Moreover, the artificial character of the experimental setting might have hindered students from engaging with it, as some students reported that they could not take all the answers seriously, as they were too hysterical. This also reveals the potential for adjusting the prompts to modify the emotional tone of the AIs, which could be addressed in future studies to examine different prompts with the aim of achieving a specific emotional tone. At the same time, the generalizability of our results is limited due to the particular learning content and setting. Future research on LLM-based AIs in authentic learning settings, implementing different content or changing the duration of the conversations, might lead to different results. In particular, research could address the question of how long or multiple instances of inducing distress in learners might affect the outcome.

## Conclusion

Our findings indicate that even within a relatively short period of interaction with an LLM-based chatbot (5–10 min), fine-tuning system prompts can intentionally elicit distinct emotions in learners. Comparing two different AI personalities by fine-tuning system prompts also revealed that chatting with an Empathic AI can elicit stronger emotions (empathy, compassion, distress) compared to chatting with a Compassionate AI. Regarding knowledge gain, we state that chatting with a generative AI chatbot instead of reading a text might not make a difference. From a practical standpoint, the challenges, such as limited control over conversational content and the chatbots’ adapted emotional tone, might inadvertently hinder knowledge acquisition. Further research is necessary to ensure reliable and contextually appropriate conversations in the context of education to foster knowledge gain.

## Supplementary Information

Below is the link to the electronic supplementary material.

Supplementary Material 1

Supplementary Material 2

Supplementary Material 3

## Data Availability

As additional source data, we have uploaded a zip-file containing the code used, a read-me file with requirements and an installation guide, and the icons used for the chat interface. The code consists of a simple python script and is includes comments to ensure easy accessibility. We made this code available via a public osf-link (https://osf.io/u3vm4/overview) and uploaded it as supplementary material 2.The source data used in this research is currently unavailable for public sharing due to strict data safety and confidentiality protocols mandated by our university. These restrictions are in place to ensure compliance with ethical standards, privacy regulations, and institutional policies.Access to the data may be granted under specific circumstances, subject to appropriate data use agreements and ethical approvals. For more information about the data, please contact the corresponding author.
