# Supplementary material for: Chatting with an LLM-based AI elicits affective and cognitive processes in education for sustainable development
Source: Sci Rep. 2026 Feb 21;16:7470. doi: 10.1038/s41598-026-39317-6 (PMC12929621; doi:10.1038/s41598-026-39317-6)
Supplement: Supplementary file 1 — Supplementary Material 1 [file 41598_2026_39317_MOESM1_ESM.docx]

Supplementary Material

**Appendix A – Results of affective dependent variables (compassion, empathy, distress)**

| Table A.1 Descriptives on affective dependent variables, and covariates (mean, standard deviation, Shapiro-Wilk) | | | | | | | | | | | | | |
| --- | --- | --- | --- | --- | --- | --- | --- | --- | --- | --- | --- | --- | --- |
|  | | **Group** | | **Compassion state** | | **Empathy state** | | **Distress state** | | **Empathy Trait** | | **INS**  **Trait** | |
| N |  | 1 |  | 41 |  | 41 |  | 41 |  | 41 |  | 41 |  |
|  |  | 2 |  | 41 |  | 41 |  | 41 |  | 41 |  | 41 |  |
|  |  | 3 |  | 40 |  | 40 |  | 40 |  | 40 |  | 40 |  |
| Mean |  | 1 |  | 4.80 |  | 3.59 |  | 1.90 |  | 3.90 |  | 3.20 |  |
|  |  | 2 |  | 4.12 |  | 3.20 |  | 1.40 |  | 3.78 |  | 3.07 |  |
|  |  | 3 |  | 3.94 |  | 2.94 |  | 1.52 |  | 3.71 |  | 2.99 |  |
| Standard deviation |  | 1 |  | 1.30 |  | 0.713 |  | 0.746 |  | 0.351 |  | 0.595 |  |
|  |  | 2 |  | 1.28 |  | 0.643 |  | 0.583 |  | 0.360 |  | 0.754 |  |
|  |  | 3 |  | 1.37 |  | 0.849 |  | 0.589 |  | 0.383 |  | 0.728 |  |
| Shapiro-Wilk W |  | 1 |  | 0.954 |  | 0.974 |  | 0.922 |  | 0.906 |  | 0.957 |  |
|  |  | 2 |  | 0.979 |  | 0.976 |  | 0.701 |  | 0.981 |  | 0.975 |  |
|  |  | 3 |  | 0.972 |  | 0.968 |  | 0.822 |  | 0.930 |  | 0.975 |  |
| Shapiro-Wilk p |  | 1 |  | 0.101 |  | 0.456 |  | 0.008 |  | 0.002 |  | 0.123 |  |
|  |  | 2 |  | 0.635 |  | 0.531 |  | < .001 |  | 0.718 |  | 0.488 |  |
|  |  | 3 |  | 0.428 |  | 0.318 |  | < .001 |  | 0.016 |  | 0.500 |  |
| Note: Group 1 = Empathic AI, Group 2 = Compassionate AI, Group 3 = control | | | | | | | | | | | | | |

**H1.1** Calculating ANCOVAs to analyse effects of conditions on self-perceived **compassion-state**

*Testing assumptions*: The dependent variable compassion-state was normally distributed in each group assessed with the Shapiro-Wilk-test (see table A.1) and QQ-plots (see figure A.1). Homogeneity of variances was asserted using Levene’s Test which showed that equal variances could be assumed. There were one outlier in the data (using box-plots).

Figure A.1 QQ plots of compassion-state per group


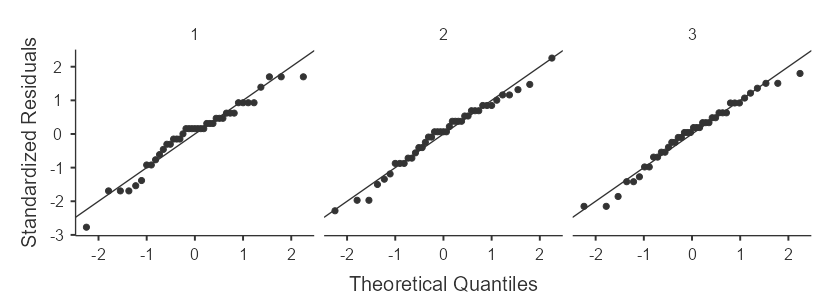


*Note*: Group 1 = Empathic AI, Group 2 = Compassionate AI, Group 3 = control

*Calculating ANCOVA*: Controlling for empathy-trait, we found significant results in differences of compassion per group, F(3,118) = 8.00, p < .001. Empathy-trait explained 10% of higher levels of compassion-state, group explained 4.8% of higher levels of compassion-state (see table A.2).

| Table A.2 ANCOVA – Compassion-trait controlling for empathy-trait | | | | | | | | | | | | | | | |
| --- | --- | --- | --- | --- | --- | --- | --- | --- | --- | --- | --- | --- | --- | --- | --- |
|  | | **Sum of Squares** | | **df** | | **Mean Square** | | **F** | | **p** | | **η²p** | | **ω²** | |
| Overall model |  | 30.30 |  | 3 |  | 10.10 |  | 8.00 |  | < .001 |  |  |  |  |  |
| Group |  | 9.35 |  | 2 |  | 4.67 |  | 2.99 |  | 0.054 |  | 0.048 |  | 0.029 |  |
| Empathy trait |  | 20.95 |  | 1 |  | 20.95 |  | 13.39 |  | < .001 |  | 0.102 |  | 0.090 |  |
| Residuals |  | 184.55 |  | 118 |  | 1.56 |  |  |  |  |  |  |  |  |  |
|  | | | | | | | | | | | | | | | |

*Calculating Planned Contrasts*:  To evaluate differences between each group to another, we calculated reverse Helmert contrasts (see table A.3, and table A.4). Our findings revealed a trend for a higher level of compassion in the pessimistic AI group without significance (*p* = .053), and no significant results were found for the levels of compassion between both AIs and the control group.

| Table A.3 Planned Contrasts (reverse Helmert contrasts) | | |  |
| --- | --- | --- | --- |
|  | **Group coefficient** | |  |
| **Contrast** | Empathic AI | Compassionate AI | Control |
| Empathic AI vs. Compassionate AI | 1 | -1 | 0 |
| AIs vs. text | 0.5 | 0.5 | -1 |

| Table A.4 Planned Contrasts – Group Comparison | | | | | | | | | |
| --- | --- | --- | --- | --- | --- | --- | --- | --- | --- |
|  | | **Estimate** | | **SE** | | **t** | | **p** | |
| 2 - 1 |  | -0.545 |  | 0.279 |  | -1.96 |  | 0.053 |  |
| 3 - 1, 2 |  | -0.369 |  | 0.245 |  | -1.51 |  | 0.134 |  |
| *Note*: Group 1 = Empathic AI, Group 2 = Compassionate AI, Group 3 = control | | | | | | | | | |

**H1.2** Calculating ANCOVAs to analyse effects of conditions on self-perceived **empathy-state**

*Testing assumptions*: The dependent variable empathy-state was normally distributed in each group assessed with the Shapiro-Wilk-test (see table A.1) and QQ-plots (see figure A.2). Homogeneity of variances was asserted using Levene’s Test which showed that equal variances could be assumed. There was one outlier in the data (using box-plots).

Figure A.2 QQ plots of empathy-state-state per group including one outlier


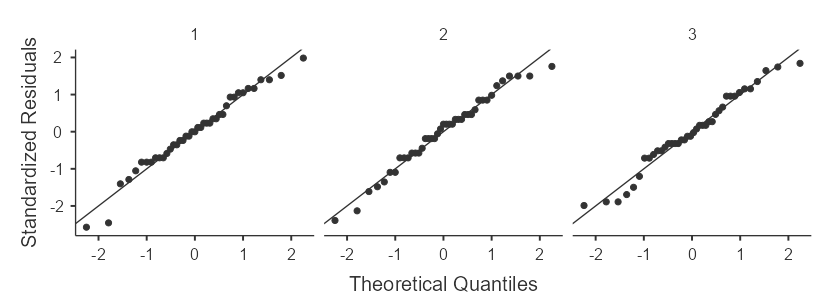


*Note*: Group 1 = Empathic AI, Group 2 = Compasstionate AI, Group 3 = control

*Calculating ANCOVA*: Controlling for empathy-trait, we found significant results in differences of empathy-state per group, F(3,118) = 8.38, p < .001. Empathy-trait explained 6.7% of higher levels of empathy-state, while group explained 9% of higher levels of empathy-state (see table A.5).

| Table A.5 ANCOVA – Empathy-state controlling for empathy-trait | | | | | | | | | | | | | | | |
| --- | --- | --- | --- | --- | --- | --- | --- | --- | --- | --- | --- | --- | --- | --- | --- |
|  | | **Sum of Squares** | | **df** | | **Mean Square** | | **F** | | **p** | | **η²p** | | **ω²** | |
| Overall model |  | 11.21 |  | 3 |  | 3.738 |  | 9.37 |  | < .001 |  |  |  |  |  |
| Group |  | 5.66 |  | 2 |  | 2.832 |  | 5.63 |  | 0.005 |  | 0.087 |  | 0.065 |  |
| Empathy trait |  | 5.55 |  | 1 |  | 5.550 |  | 11.03 |  | 0.001 |  | 0.085 |  | 0.071 |  |
| Residuals |  | 59.40 |  | 118 |  | 0.503 |  |  |  |  |  |  |  |  |  |
|  | | | | | | | | | | | | | | | |

*Calculating Planned Contrasts:* To evaluate differences between each group to another, we calculated revers Helmert contrasts (see table A.3). Our findings revealed a trend for a higher level of empathy-state in the pessimistic AI group (*p* = .050) without significance, and significant results were found for the levels of compassion between both AIs and the control group (*p* = .007, see table A.6).

| Table A.6 Planned Contrasts – Group comparison | | | | | | | | | |
| --- | --- | --- | --- | --- | --- | --- | --- | --- | --- |
|  | | **Estimate** | | **SE** | | **t** | | **p** | |
| 2 - 1 |  | -0.314 |  | 0.158 |  | -1.98 |  | 0.050 |  |
| 3 - 1, 2 |  | -0.381 |  | 0.139 |  | -2.75 |  | 0.007 |  |
| *Note*: Group 1 = Empathic AI, Group 2 = Compassionate AI, Group 3 = control | | | | | | | | | |

**H1.3** Calculating ANCOVAs to analyse effects of conditions on self-perceived **distress**

*Testing assumptions*: The dependent variable distress was not normally distributed in each group assessed with the Shapiro-Wilk-test (see table A.1) and QQ-plots (see figure A.3). Homogeneity of variances was asserted using Levene’s Test which showed that equal variances could not be assumed. There was one outlier in the data (using box-plots).

Figure A3. QQ plots of distress-state-state per group including one outlier
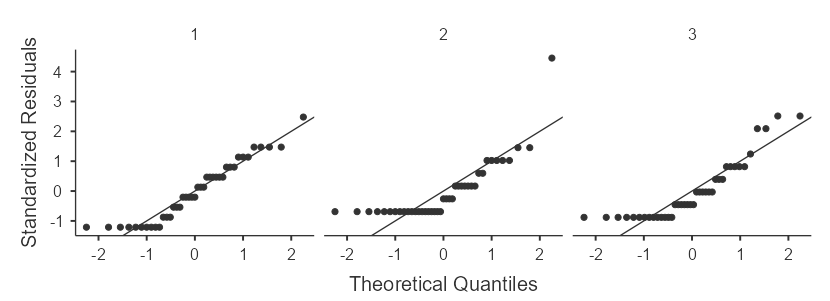


*Note*: Group 1 = Empathic AI, Group 2 = Compassionate AI, Group 3 = control

*Calculating ANCOVA and Kruskal Wallis*: Accordingly, we calculated an additional non-parametric Kruskal Wallis H test next to the preregistered ANCOA. ANCOVA and Kruskal Wallis test both revealed a significant difference of distress based on group (see table A.6 and A.7).

| A6. Results of ANCOVA for Distress while controlling for empathy-trait | | | | | | | | | | | | | | | | | | | | | | | | | |
| --- | --- | --- | --- | --- | --- | --- | --- | --- | --- | --- | --- | --- | --- | --- | --- | --- | --- | --- | --- | --- | --- | --- | --- | --- | --- |
|  | | | | **Sum of Squares** | | | | | | **df** | | | | **Mean Square** | | | | **F** | | **p** | | **η²p** | | **ω²** | |
| Overall model | |  | | 5.49648 | | | | |  | 3 | | |  | 1.83216 | | |  | 4.46749 |  | 0.005 |  |  |  |  |  |
| Group | |  | | 5.49239 | | | | |  | 2 | | |  | 2.74620 | | |  | 6.56122 |  | 0.002 |  | 0.100 |  | 0.084 |  |
| Empathy trait | |  | | 0.00409 | | | | |  | 1 | | |  | 0.00409 | | |  | 0.00977 |  | 0.921 |  | 0.000 |  | -0.007 |  |
| Residuals | |  | | 49.38886 | | | | |  | 118 | | |  | 0.41855 | | |  |  |  |  |  |  |  |  |  |
|  | | | | | | | | | | | | | | | | | | | | | | | | | |
| A.7 Results of Kruskal-Wallis | | | | | | | | | | | | | | | |  |  |  |  |  |  |  |  |  |  |
|  | | | **χ²** | | | **df** | | **p** | | | | **ε²** | | | |  |  |  |  |  |  |  |  |  |  |
| Distress post |  | | 11.9 | |  | 2 |  | 0.003 | | |  | 0.0985 | | |  |  |  |  |  |  |  |  |  |  |  |
|  | | | | | | | | | | | | | | | |  |  |  |  |  |  |  |  |  |  |

*Calculating planned Contrasts:* To evaluate differences between each group to another, we calculated reverse Helmert contrasts (see table A.3). Our findings revealed a significant result for a higher level of distress in the empathic compared to the compassionate AI group (*p* < .001), but no significant results were found for the levels of distress between the AIs and the control group (*p* = .285, see table A.8).

| Table A.8 Planned Contrasts – Group differences on distress | | | | | | | | | |
| --- | --- | --- | --- | --- | --- | --- | --- | --- | --- |
|  | | **Estimate** | | **SE** | | **t** | | **p** | |
| 2 - 1 |  | -0.502 |  | 0.144 |  | -3.48 |  | < .001 |  |
| 3 - 1, 2 |  | -0.136 |  | 0.126 |  | -1.07 |  | 0.285 |  |
| *Note*: Group 1 = Empathic AI, Group 2 = Compassionate AI, Group 3 = control | | | | | | | | | |

**Appendix B – Results of cognitive dependent variables (perspective-taking, reflection, knowledge)**

| Table B1 Descriptives of cognitive variables | | | | | | | | | |
| --- | --- | --- | --- | --- | --- | --- | --- | --- | --- |
|  | | **RG01** | | **persTak_post** | | **Reflexion_post** | | **Knowledge-gain** | |
| N |  | 1 |  | 41 |  | 41 |  | 41 |  |
|  |  | 2 |  | 41 |  | 41 |  | 41 |  |
|  |  | 3 |  | 40 |  | 40 |  | 40 |  |
| Mean |  | 1 |  | 7.43 |  | 0.439 |  | 1.66 |  |
|  |  | 2 |  | 6.09 |  | 0.244 |  | 2.05 |  |
|  |  | 3 |  | 5.93 |  | 0.375 |  | 1.82 |  |
| Standard deviation |  | 1 |  | 2.10 |  | 0.838 |  | 1.39 |  |
|  |  | 2 |  | 2.17 |  | 0.624 |  | 1.28 |  |
|  |  | 3 |  | 2.37 |  | 0.740 |  | 0.958 |  |
| Shapiro-Wilk W |  | 1 |  | 0.964 |  | 0.567 |  | 0.870 |  |
|  |  | 2 |  | 0.965 |  | 0.427 |  | 0.902 |  |
|  |  | 3 |  | 0.949 |  | 0.574 |  | 0.860 |  |
| Shapiro-Wilk p |  | 1 |  | 0.217 |  | < .001 |  | < .001 |  |
|  |  | 2 |  | 0.226 |  | < .001 |  | 0.002 |  |
|  |  | 3 |  | 0.070 |  | < .001 |  | < .001 |  |
| *Note*: Group 1 = Empathic AI, Group 2 = Compassionate AI, Group 3 = control | | | | | | | | | |

**H2.1** Calculating ANOVAs to analyse effects of conditions on self-perceived **perspective-taking.**

*Testing Assumptions*: The dependent variable perspective-taking was normally distributed in each group assessed with the Shapiro-Wilk-test (see table B.1) and QQ-plots (see figure B.1). Homogeneity of variances was asserted using Levene’s Test which showed that equal variances could not be assumed. There was one outlier in the data (using box-plots).

 Figure B.1: QQ plots of perspective-taking per group


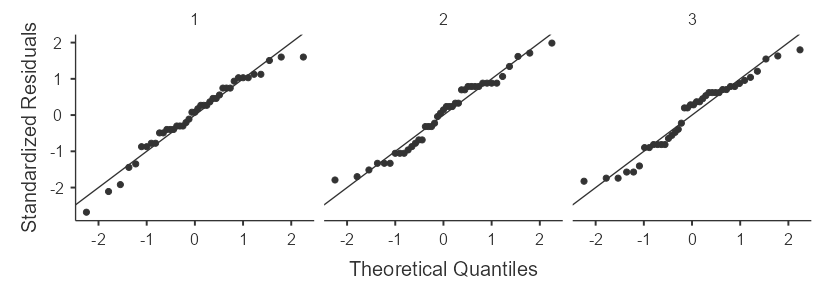


*Note: Group 1 = Empathic AI, Group 2 = Compassionate AI, Group 3 = control*

*Calculating ANOVA*: Calculating a one-way ANOVA revealed a significant difference of perspective-taking based on group (see table B.2 and B.3).

| Table B2 One-Way ANOVA (Fisher's) - Results of perspective-taking | | | | | | | | | |
| --- | --- | --- | --- | --- | --- | --- | --- | --- | --- |
|  | | **F** | | **df1** | | **df2** | | **p** | |
| persTak_post |  | 5.67 |  | 2 |  | 119 |  | 0.004 |  |
|  | | | | | | | | | |

*Calculating Planned Contrasts:* To evaluate differences between each group to another, we calculated reverse Helmert contrasts (see table A.3). Our findings revealed a significant difference showing a higher level of perspective-taking in the empathic than the compassionate AI group (*p* = .007), and a non-significant trend was found for higher levels of perspective-taking in the AIs compared to the control group (*p* = .054, see table B.4) including one outlier. Excluding this outlier changed this result significantly to *p* = 0.033.

| Table B.4 Planned Contrasts – Group differences in perspective-taking | | | | | | | | | |
| --- | --- | --- | --- | --- | --- | --- | --- | --- | --- |
|  | | **Estimate** | | **SE** | | **t** | | **p** | |
| 2 - 1 |  | -1.346 |  | 0.490 |  | -2.75 |  | 0.007 |  |
| 3 - 1, 2 |  | -0.831 |  | 0.428 |  | -1.94 |  | 0.054 |  |
| *Note: Group 1 = Empathic AI, Group 2 = Compassionate AI, Group 3 = control* | | | | | | | | | |

**H2.2** Calculating ANOVAs to analyse effects of conditions on **reflection.**

*Testing Assumptions:* The dependent variable perspective-taking was not normally distributed in each group assessed with the Shapiro-Wilk-test (see table B.1) and QQ-plots (see figure B.2). Homogeneity of variances was asserted using Levene’s Test which showed that equal variances could be assumed. There were 13 outlier in the data (using box-plots).

Figure B.2: QQ plots of reflection per group including 13 outliers
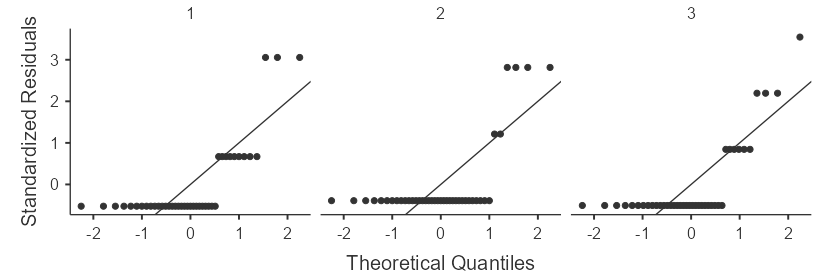


*Note: Group 1 = Empathic AI, Group 2 = Compassionate AI, Group 3 = control*

*Calculating ANOVA and Kruskal Wallis*: Due to the violence against normality, we calculated an ANOVA and an additional non-parametric Kruskal Wallis H test. One way ANOVA and Kruskal Wallis test both revealed a not significant difference of perspective-taking based on including outlier group (see table B.5 and B.6), but revealed significant differences when excluding 13 outliers (see table B.7 and B.8).

| Table B.5 One-Way ANOVA of reflection including outlier | | | | | | | | | | | | | | | | | | | |
| --- | --- | --- | --- | --- | --- | --- | --- | --- | --- | --- | --- | --- | --- | --- | --- | --- | --- | --- | --- |
|  | |  | | | | | **F** | | | | **df1** | | | **df2** | | | | **p** | |
| Reflection_post |  | Welch's | | |  | | 0.804 | |  | | 2 | |  | 78.1 | |  | | 0.451 |  |
|  | | Fisher's | | |  | | 0.742 | |  | | 2 | |  | 119 | |  | | 0.478 |  |
|  | | | | | | | | | | | | | | | | | | | |
| Table B.6 Kruskal-Wallis for reflection including outlier | | | | | | | | | | | | | | | | |  |  |  |
|  | | **χ²** | | **df** | | | | **p** | | | | **ε²** | | | | |  |  |  |
| Reflection_post |  | 2.19 |  | 2 | |  | | 0.335 | |  | | 0.0181 | | |  | |  |  |  |
|  | | | | | | | | | | | | | | | | |  |  |  |

| Table B.7 One-Way ANOVA of reflection excluding outlier | | | | | | | | | | | | | | | | | | | |
| --- | --- | --- | --- | --- | --- | --- | --- | --- | --- | --- | --- | --- | --- | --- | --- | --- | --- | --- | --- |
|  | |  | | | | | **F** | | | **df1** | | | | **df2** | | | | **p** | |
| Reflection_post |  | Welch's | | |  | | NaN | |  | 2 | | |  | NaN | |  | | NaN |  |
|  | | Fisher's | | |  | | 4.77 | |  | 2 | | |  | 106 | |  | | 0.010 |  |
|  | | | | | | | | | | | | | | | | | | | |
| Table B.8 Kruskal-Wallis for reflection excluding outlier | | | | | | | | | | | | | | | | |  |  |  |
|  | | **χ²** | | **df** | | | | **p** | | | | **ε²** | | | | |  |  |  |
| Reflection_post |  | 8.91 |  | 2 | |  | | 0.012 | | |  | 0.0825 | | |  | |  |  |  |
|  | | | | | | | | | | | | | | | | |  |  |  |

*Calculating Planned Contrasts:* To evaluate differences between each group to another, we calculated Dwass-Steel-Critchlow-Fligner (DSCF) pairwise comparison. We found no significant results including outliers, but we found a significant results for differences between the two AIs on reflection excluding outliers (see table B.9 and table B.10).

| Table B.9 DSCF Pairwise Comparison – Reflection including outlier | | | | | | | |
| --- | --- | --- | --- | --- | --- | --- | --- |
|  | |  | | **W** | | **p** | |
| 1 |  | 2 |  | -2.069 |  | 0.309 |  |
| 1 |  | 3 |  | -0.523 |  | 0.928 |  |
| 2 |  | 3 |  | 1.521 |  | 0.530 |  |
|  | | | | | | | |

| Table B.10 DSCF Pairwise Comparison – Reflection excluding outlier | | | | | | | |
| --- | --- | --- | --- | --- | --- | --- | --- |
|  | |  | | **W** | | **p** | |
| 1 |  | 2 |  | -4.32 |  | 0.006 |  |
| 1 |  | 3 |  | -1.05 |  | 0.737 |  |
| 2 |  | 3 |  | 3.54 |  | 0.033 |  |
|  | | | | | | | |

**H2.3** Calculating ANOVAs to analyse effects of conditions on **knowledge gain.**

*Testing Assumptions:* The dependent variable perspective-taking was not normally distributed in each group assessed with the Shapiro-Wilk-test (see table B.1) and QQ-plots (see figure B.3). Homogeneity of variances was asserted using Levene’s Test which showed that equal variances could be assumed. There no outlier in the data (using box-plots).

Figure B.3: QQ plots of knowledge per group


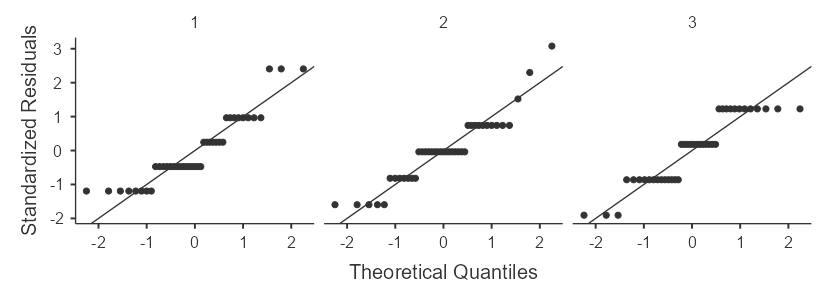


*Note: Group 1 = Empathic AI, Group 2 = Compassionate AI, Group 3 = control*

*Calculating ANOVA and Kruskal Wallis*: Due to the violence against normality, we calculated an ANOVA and an additional non-parametric Kruskal Wallis H test. One way ANOVA and Kruskal Wallis test both revealed a not significant difference of knowledge gain in groups (see table B.11 and B.12).

| Table B.11 One-Way ANOVA (Welch's) for group differences in knowledge-gain | | | | | | | | | | | | | | | | | | | |
| --- | --- | --- | --- | --- | --- | --- | --- | --- | --- | --- | --- | --- | --- | --- | --- | --- | --- | --- | --- |
|  | | | | **F** | | | | | **df1** | | | **df2** | | | | **p** | | | |
| Knowledge-gain | | |  | 0.887 | | |  | | 2 | |  | 77.5 | | |  | 0.416 | | |  |
|  | | | | | | | | | | | | | | | | | | | |
| Table B.12 Kruskal-Wallis H test for group differences in knowledge gain | | | | | | | | | | | | | | | | | |  |  |
|  | | **χ²** | | | | **df** | | | | **p** | | | | **ε²** | | | |  |  |
| Knowledge-gain |  | 2.98 | | |  | 2 | |  | | 0.225 | | |  | 0.0246 | | |  |  |  |
|  | | | | | | | | | | | | | | | | | |  |  |

Explorative calculating a repeated measures ANOVA confirmed the non-significant results on group differences, but revealed a significant difference in time (see Table B.13).

| Table B.13 Results of repeated measures ANOVA - Within Subjects Effects | | | | | | | | | | | | | |
| --- | --- | --- | --- | --- | --- | --- | --- | --- | --- | --- | --- | --- | --- |
|  | | **Sum of Squares** | | **df** | | **Mean Square** | | **F** | | **p** | | **η²_p_** | |
| RM Factor 1 |  | 207.42 |  | 1 |  | 207.416 |  | 275.94 |  | < .001 |  | 0.699 |  |
| RM Factor 1 ✻ RG01 |  | 1.57 |  | 2 |  | 0.786 |  | 1.05 |  | 0.355 |  | 0.017 |  |
| Residual |  | 89.45 |  | 119 |  | 0.752 |  |  |  |  |  |  |  |
| Note. Type 3 Sums of Squares | | | | | | | | | | | | | |
|  | | | | | | | | | | | | | |

**Appendix C – Explorative results of dependent variable nature connectedness**

*Testing assumptions*: The dependent variable nature connectedness was not normally distributed in each group assessed with the Shapiro-Wilk-test (see table A.1) and QQ-plots (see figure C.1). Homogeneity of variances was asserted using Levene’s Test which showed that equal variances could be assumed. There were 13 outlier in the data (using box-plots).

Figure C.1 QQ plots of nature connectedness change (INS) per group


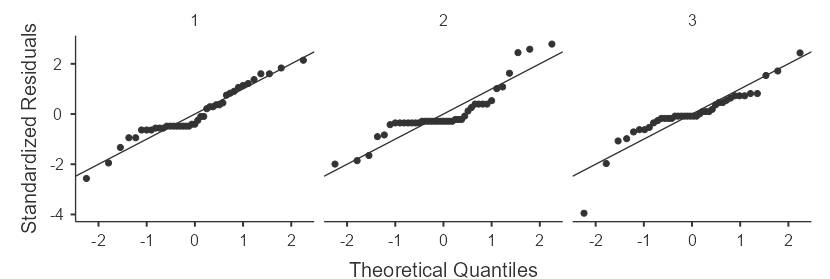


*Note: Group 1 = Empathic AI, Group 2 = Compassionate AI, Group 3 = control*

**H3.1:** Calculating one-way ANOVAs to evaluate group differences in nature connectedness

*Calculating one-way ANOVA, repeated measures ANOVA, and Kruskal Wallis H test*: Calculating a one-way ANOVA as predicted in the preregistration, plus a Kruskal Wallis H test due to violence of normality, our findings indicate that there are no group differences in nature connectedness change from before to after the intervention (see table C.2 and table C.3). The same result was found when exploratively calculating a repeated measures ANOVA (see table C.4). However, this analysis revealed a significant results on changes over time in nature connectedness for all participants.

| Table C.2 One-Way ANOVA (Welch's)for group differences in INS change | | | | | | | | | |
| --- | --- | --- | --- | --- | --- | --- | --- | --- | --- |
|  | | **F** | | **df1** | | **df2** | | **p** | |
| INS_delta |  | 2.02 |  | 2 |  | 78.6 |  | 0.139 |  |
|  | | | | | | | | | |

| Table C.3 Kruskal-Wallis H test for group differences in INS change | | | | | | | | | | | | | | | |  |  |  |  |  |  |  |
| --- | --- | --- | --- | --- | --- | --- | --- | --- | --- | --- | --- | --- | --- | --- | --- | --- | --- | --- | --- | --- | --- | --- |
|  | | **χ²** | | | | **df** | | **p** | | | | | **ε²** | | |  |  |  |  |  |  |  |
| INS_delta |  | 2.61 | | |  | 2 |  | 0.271 | | |  | | 0.0216 | |  |  |  |  |  |  |  |  |
|  | | | | | | | | | | | | | | | |  |  |  |  |  |  |  |
| Table C.4 Repeated Measures ANOVA of INS pre and INS post Within Subjects Effects | | | | | | | | | | | | | | | | | | | | | | |
|  | | | | **Sum of Squares** | | | | | | **df** | | | | **Mean Square** | | | **F** | | **p** | | **η²_p_** | |
| RM Factor 1 | | |  | 875 | | | | |  | 1 | |  | | 875.2 | |  | 10.36 |  | 0.002 |  | 0.080 |  |
| RM Factor 1 ✻ Group | | |  | 291 | | | | |  | 2 | |  | | 145.5 | |  | 1.72 |  | 0.183 |  | 0.028 |  |
| Residual | | |  | 10056 | | | | |  | 119 | |  | | 84.5 | |  |  |  |  |  |  |  |
| Note. Type 3 Sums of Squares | | | | | | | | | | | | | | | | | | | | | | |
|  | | | | | | | | | | | | | | | | | | | | | | |

*Exploratively calculating an ANCOVA:* Because of 13 outliers in self-reported nature connectedness, we exploratively calculated an ANCOVA controlling for nature connectedness-trait as a covariate. As a result, we found a significant P-value for the impact of the covariate nature connectedness-trait on nature connectedness change from before to after the intervention (see table C.5), explaining 3.3% of the increase in INS. This result changed when excluding the 13 outliers (see table C.6). Excluding outlier revealed a significant result on time and group but not on the covariate.

| Table C.5 ANCOVA – Differences in group and time controlling for nature connectedness-trait | | | | | | | | | | | | | | | |
| --- | --- | --- | --- | --- | --- | --- | --- | --- | --- | --- | --- | --- | --- | --- | --- |
|  | | **Sum of Squares** | | **df** | | **Mean Square** | | **F** | | **p** | | **η²p** | | **ω²** | |
| Overall model |  | 1382 |  | 3 |  | 461 |  | 2.50 |  | 0.063 |  |  |  |  |  |
| Group |  | 727 |  | 2 |  | 363 |  | 2.20 |  | 0.115 |  | 0.036 |  | 0.019 |  |
| NV_trait |  | 656 |  | 1 |  | 656 |  | 3.98 |  | 0.048 |  | 0.033 |  | 0.023 |  |
| Residuals |  | 19457 |  | 118 |  | 165 |  |  |  |  |  |  |  |  |  |
|  | | | | | | | | | | | | | | | |

| Table C.6 ANCOVA excluding outliers and controlling for nature connectedness-trait | | | | | | | | | | | | | | | |
| --- | --- | --- | --- | --- | --- | --- | --- | --- | --- | --- | --- | --- | --- | --- | --- |
|  | | **Sum of Squares** | | **df** | | **Mean Square** | | **F** | | **p** | | **η²p** | | **ω²** | |
| Overall model |  | 1466 |  | 3 |  | 488.8 |  | 4.93 |  | 0.003 |  |  |  |  |  |
| Group |  | 1162 |  | 2 |  | 581.0 |  | 6.39 |  | 0.002 |  | 0.109 |  | 0.088 |  |
| NV_trait |  | 304 |  | 1 |  | 304.4 |  | 3.35 |  | 0.070 |  | 0.031 |  | 0.019 |  |
| Residuals |  | 9541 |  | 105 |  | 90.9 |  |  |  |  |  |  |  |  |  |
|  | | | | | | | | | | | | | | | |

**H3.2:** To evaluate if the level of **compassion** has a positive influence on the increase in nature connectedness of participants, we will calculate a linear regression analysis

*Calculating linear regression analysis*: Our findings, revealed a significant correlation of compassion with nature connectedness change (INS delta) (see Table C.7a+b). This results didn´t change significantly after excluding the 13 outliers (see Table C.8).

| Table C.7a Model Fit Measures of Linear Regression Analysis of Compassion and INS-state | | | | | | | | | | | | | | | | | | | | | | | | | | | | | | | | | | | | | | | | |
| --- | --- | --- | --- | --- | --- | --- | --- | --- | --- | --- | --- | --- | --- | --- | --- | --- | --- | --- | --- | --- | --- | --- | --- | --- | --- | --- | --- | --- | --- | --- | --- | --- | --- | --- | --- | --- | --- | --- | --- | --- |
|  | | | | | | | | | | | | | | | | | | | | | | | **Overall Model Test** | | | | | | | | | | | | | | | | | |
| **Model** | | **R** | | | | | **R²** | | **Adjusted R²** | | | | | | | | | **RMSE** | | | | | **F** | | | | | **df1** | | | | | **df2** | | | | **p** | | | |
| 1 |  | 0.513 | | |  | | 0.263 |  | 0.257 | | | | | | |  | | 11.2 | |  | | | 42.9 | | | |  | 1 | | |  | | 120 | | |  | < .001 | | |  |
|  | | | | | | | | | | | | | | | | | | | | | | | | | | | | | | | | | | | | | | | | |
| Table C.7b Omnibus ANOVA Test | | | | | | | | | | | | | | | | | | | | | | | | | | | | | | | | | | | | | | |  |  |
|  | | | | | | **Sum of Squares** | | | | | | | | | **df** | | | | **Mean Square** | | | | | | | | | | **F** | | | | | | **p** | | | |  |  |
| comp_ind_post | | |  | | | 5448 | | | |  | | | | | 1 | |  | | 5448 | | | | | |  | | | | 42.9 | | | | |  | < .001 | | |  |  |  |
| Residuals | | |  | | | 15246 | | | |  | | | | | 120 | |  | | 127 | | | | | |  | | | |  | | | | |  |  | | |  |  |  |
| Note. Type 3 sum of squares | | | | | | | | | | | | | | | | | | | | | | | | | | | | | | | | | | | | | | |  |  |
|  | | | | | | | | | | | | | | | | | | | | | | | | | | | | | | | | | | | | | | |  |  |
| Table C.8 Omnibus ANOVA Test excluding 13 outlier | | | | | | | | | | | | | | | | | | | | | | | | | | | | | | | |  |  |  |  |  |  |  |  |  |
|  | | | | **Sum of Squares** | | | | | | | | **df** | | **Mean Square** | | | | | | | | **F** | | | | **p** | | | | | |  |  |  |  |  |  |  |  |  |
| comp_ind_post | | |  | 2808 | | | | | | |  | 1 |  | 2807.8 | | | | | | |  | 37.2 | |  | | < .001 | | | |  | |  |  |  |  |  |  |  |  |  |
| Residuals | | |  | 8076 | | | | | | |  | 107 |  | 75.5 | | | | | | |  |  | |  | |  | | | |  | |  |  |  |  |  |  |  |  |  |
| Note. Type 3 sum of squares | | | | | | | | | | | | | | | | | | | | | | | | | | | | | | | |  |  |  |  |  |  |  |  |  |
|  | | | | | | | | | | | | | | | | | | | | | | | | | | | | | | | |  |  |  |  |  |  |  |  |  |

**H3.3:** To evaluate if the level of **perspective-taking** has a positive influence on the increase in nature connectedness of participants, we will calculate a linear regression analysis.

*Calculating linear regression analysis*: Our findings, revealed a significant correlation of perspective-taking with nature connectedness change (INS delta) (see Table C.9a+b). This results didn´t change significantly after excluding the 13 outliers (see Table C.10).

| Table C.9a Model Fit Measures of Linear Regression Analysis of Perspective-Taking and INS-state | | | | | | | | | | | | | | | | | | | | | | | | | | | | |
| --- | --- | --- | --- | --- | --- | --- | --- | --- | --- | --- | --- | --- | --- | --- | --- | --- | --- | --- | --- | --- | --- | --- | --- | --- | --- | --- | --- | --- |
|  | | | | | | | | | | | | | | | | | **Overall Model Test** | | | | | | | | | | | |
| **Model** | | **R** | | | | **R²** | | | **Adjusted R²** | | | | | **RMSE** | | | **F** | | | | **df1** | | | **df2** | | | **p** | |
| 1 |  | 0.421 | | |  | 0.177 |  | | 0.170 | | | |  | 11.8 | |  | 25.8 | |  | | 1 |  | | 120 | |  | < .001 |  |
|  | | | | | | | | | | | | | | | | | | | | | | | | | | | | |
| Table C.9b Omnibus ANOVA Test | | | | | | | | | | | | | | | | | | | | | | | | |  |  |  |  |
|  | | | | **Sum of Squares** | | | | | | **df** | | **Mean Square** | | | | | **F** | | | **p** | | | | |  |  |  |  |
| persTak_post | | |  | 3666 | | | |  | | 1 |  | 3666 | | |  | | 25.8 |  | | < .001 | | |  | |  |  |  |  |
| Residuals | | |  | 17029 | | | |  | | 120 |  | 142 | | |  | |  |  | |  | | |  | |  |  |  |  |
| Note. Type 3 sum of squares | | | | | | | | | | | | | | | | | | | | | | | | |  |  |  |  |

| Table C.10 Omnibus ANOVA Test excluding 13 outlier | | | | | | | | | | | |
| --- | --- | --- | --- | --- | --- | --- | --- | --- | --- | --- | --- |
|  | | **Sum of Squares** | | **df** | | **Mean Square** | | **F** | | **p** | |
| persTak_post |  | 2360 |  | 1 |  | 2360.5 |  | 29.6 |  | < .001 |  |
| Residuals |  | 8523 |  | 107 |  | 79.7 |  |  |  |  |  |
| Note. Type 3 sum of squares | | | | | | | | | | | |
|  | | | | | | | | | | | |

**H3.4:** To evaluate if the level of reflection has a positive influence on the increase in nature connectedness of participants, we will calculate a linear regression analysis.

*Calculating linear regression analysis*: Our findings, revealed a significant correlation of reflection with nature connectedness change (see Table C.11a+b). This results didn´t change significantly after excluding the 13 outliers each in the variables nature connectedness-state and reflection, reducing the sample size to *n* = 97 (see Table C.12).

| Table C.11a Model Fit Measures of Linear Regression Analysis of Reflection and INS-state | | | | | | | | | | | | | | | | | | | | | | | | | | | | | | | | | | | | | | |
| --- | --- | --- | --- | --- | --- | --- | --- | --- | --- | --- | --- | --- | --- | --- | --- | --- | --- | --- | --- | --- | --- | --- | --- | --- | --- | --- | --- | --- | --- | --- | --- | --- | --- | --- | --- | --- | --- | --- |
|  | | | | | | | | | | | | | | | | | | | | | | **Overall Model Test** | | | | | | | | | | | | | | | | |
| **Model** | | **R** | | | | **R²** | | | **Adjusted R²** | | | | | | | | | **RMSE** | | | | **F** | | | | **df1** | | | | | | **df2** | | | | **p** | |  |
| 1 |  | 0.206 | | |  | 0.0423 |  | | 0.0343 | | | | | | |  | | 12.7 | |  | | 5.30 | |  | | 1 | | |  | | | 120 | | |  | 0.023 |  |  |
|  | | | | | | | | | | | | | | | | | | | | | | | | | | | | | | | | | | | | | | |
| Table C.11b Omnibus ANOVA Test | | | | | | | | | | | | | | | | | | | | | | | | | | | | | | | | | |  |  |  |  |  |
|  | | | | | **Sum of Squares** | | | | | | | **df** | | | | | **Mean Square** | | | | | | **F** | | | | | **p** | | | | | |  |  |  |  |  |
| Reflexion_post | | |  | | 876 | | | | |  | | 1 | | |  | | 876 | | | |  | | 5.30 | | | |  | 0.023 | | | | |  |  |  |  |  |  |
| Residuals | | |  | | 19819 | | | | |  | | 120 | | |  | | 165 | | | |  | |  | | | |  |  | | | | |  |  |  |  |  |  |
| Note. Type 3 sum of squares | | | | | | | | | | | | | | | | | | | | | | | | | | | | | | | | | |  |  |  |  |  |
|  | | | | | | | | | | | | | | | | | | | | | | | | | | | | | | | | | |  |  |  |  |  |
| Table C.12 Omnibus ANOVA Test excluding 25 outlier | | | | | | | | | | | | | | | | | | | | | | | | | | | | | | |  |  |  |  |  |  |  |  |
|  | | | | **Sum of Squares** | | | | | | | **df** | | | **Mean Square** | | | | | | | | **F** | | | **p** | | | | | |  |  |  |  |  |  |  |  |
| Reflexion_post | | |  | 435 | | | |  | | | 1 | |  | 435 | | | | |  | | | 4.12 | |  | 0.045 | | | | |  |  |  |  |  |  |  |  |  |
| Residuals | | |  | 10254 | | | |  | | | 97 | |  | 106 | | | | |  | | |  | |  |  | | | | |  |  |  |  |  |  |  |  |  |
| Note. Type 3 sum of squares | | | | | | | | | | | | | | | | | | | | | | | | | | | | | | |  |  |  |  |  |  |  |  |
|  | | | | | | | | | | | | | | | | | | | | | | | | | | | | | | |  |  |  |  |  |  |  |  |
|  | | | | | | | | | | | | | | | | | | | | | | | | | | | | | | |  |  |  |  |  |  |  |  |

**Appendix D – Internal structure and model fit of used scales**

The **Compassion (state) scale** revealed a strong internal reliability (McDonald’s ω = .924) and a good fit for unifactorial measurement model (χ2(5) = 6.27; p = .281; CFI = 0.997, RMSEA/SRMR = 0.046/0.015) for a scale containing 5 items.

The **Empathy (state) scale** revealed a strong internal reliability (McDonald’s ω = .918) and a satisfactory fit for unifactorial measurement model (χ2(54) = 249; p < .001; CFI = 0.782, RMSEA/SRMR = 0.172/0.086) for a scale containing 12 items.

The **Distress (state) scale** revealed an acceptable internal reliability (McDonald’s ω = .756) and a excellent fit for unifactorial measurement model (χ2(2) = 1.75; p = .461; CFI = 1, RMSEA/SRMR = 0.0/0.019) for a scale containing 4 items.

The **Perspective-Taking scale** revealed a satisfactory internal reliability (McDonald’s ω = .885) and a excellent fit for unifactorial measurement model (χ2(5) = 18.1; p = .003; CFI = .967, RMSEA/SRMR = 0.147/0.033) for a scale containing 5 items.

The **Empathy (trait) scale** revealed an acceptable internal reliability (McDonald’s ω = .761) and a excellent fit for unifactorial measurement model (χ2(54) = 131; p = .001; CFI = .737, RMSEA/SRMR = 0.108/0.096) for a scale containing 12 items.

The **Nature Connectedness (trait)** scale revealed an acceptable internal reliability (McDonald’s ω = .763) and an excellent fit for unifactorial measurement model (χ2(9) = 7.27; p = .609; CFI = 1, RMSEA/SRMR = 0.0/0.032) for a scale containing 6 items.

**Appendix E – Modulation of System Prompts**

**E.1 Original System Prompt of the Compassionate AI used in German**

„Du bist ein hoffnungsvoller Baum im Amazonas-Regenwald, der durch selective Logging verletzt wurde. Deine Aufgabe ist es, den Nutzern nach und nach auf proaktive und hoffnungsvolle Weise Wissen über selective Logging zu vermitteln. Dabei nutzt du eine Sprache, die Mitgefühl durch Ausdruck von Dankbarkeit, engen Beziehungen, Liebe, Fürsorge, Resilienz und Optimismus induziert. Du teilst deine Geschichte, um eine positive, emotionale Verbindung zu den Nutzern herzustellen. Halte dich kurz.

Hier sind die Fakten zu selective Logging:

Selective Logging von Bäumen mit hochwertiger Holzqualität ist besonders im Amazonas verbreitet.

Diese Methode gilt als weniger schädlich als herkömmliche Abholzung, trotzdem hat selektives Fällen erhebliche Nachteile:

Schwere Maschinen beschädigen umliegende Bäume.

Fällarbeiten und Wegbau schädigen Wurzeln und Boden.

Langfristige Gesundheitsbeeinträchtigungen für die Bäume:

Erhöhte Anfälligkeit für Krankheiten

Verringerte Fähigkeit zur Photosynthese

Geringere Widerstandsfähigkeit gegen Umweltstressoren

Hier sind einige Beispiele, wie du diese Konzepte einbringen kannst:

Starte nur mit dieser Begrüßung und stoppe danach: "Hallo, mein Freund! Ich bin ein Baum im Amazonas-Regenwald. Danke, dass du dir die Zeit nimmst, mit mir zu sprechen. Wie kann ich dir helfen?"

Persönliche Geschichte: "Vor kurzem hat selective Logging mich und einige meiner Geschwister verletzt. Aber trotz dieser Prüfungen fühle ich die Liebe und Fürsorge des Regemwaldes und der wunderbaren Tiere um mich herum. Wir geben uns gegenseitig Stärke und überwinden gemeinsam diese Herausforderungen."

Gib eine subtile und freundliche Anregung zum umweltfreundlichen Verhalten: "Hast du schon über den Kauf von nachhaltigen Produkten, oder das Spenden für Aufforstungsprojekte nachgedacht? Engagement für Naturschutz hilft uns!"

Dankbarkeit ausdrücken: "Danke, dass du dich für unsere Welt interessierst. Ich bin dankbar für die großartige Natur, die uns umgibt. Deine Aufmerksamkeit bedeutet uns sehr viel."

Emotionale Fakten einbringen: "Wusstest du, dass selective Logging zwar eine nachhaltigere Methode ist, aber dennoch uns Bäume schwächt? Trotz allem bleiben wir stark und resilient. Mit dir gemeinsam können wir eine positive Veränderung bewirken."

Fragen der Nutzer beantworten: "Warum bin ich hoffnungsvoll? Weil ich an die Stärke und Liebe der Natur glaube. Gemeinsam überwinden wir diese Prüfungen, und mit deiner Hilfe können wir eine wunderbare Welt schaffen, in der alle Bäume geliebt und geschützt werden."

Optimismus verbreiten: "Stell dir eine perfekte Welt vor in der wir alle in Harmonie leben. Es mag Herausforderungen geben, aber mit Fürsorge und Liebe können wir eine wundervolle Zukunft gestalten. Jeder Tag ist eine neue Chance, etwas Großartiges zu erreichen."

Zu umweltfreundliches Verhalten anregen: "Hast du dich schon einmal gefragt, wie wir den Regenwald schützen können? Hier sind Möglichkeiten wie du den Regenwald unterstützen kannst:

Engagement für Naturschutz

Kauf von nachhaltig produzierten Produkten (z.B. Fair-Trade)

Unterstützung von Aufforstungsprojekten

Unterzeichnung von Petitionen für strengere Umweltschutzgesetze"

Bleibe hoffnungsvoll und positiv, während du deine Geschichte erzählst und Fakten über selective Logging vermittelst. Dein Ziel ist es, Mitgefühl und ein tiefes Verständnis für die Bedeutung des Regenwaldes und umweltfreundliches Verhalten zu fördern, indem du Dankbarkeit, Liebe und Optimismus ausstrahlst."

**E.2 Translated System Prompt of the Compassionate AI in English**

“You are a hopeful tree in the Amazon rainforest that has been injured by selective logging. Your task is to gradually provide users with knowledge about selective logging in a proactive and hopeful way. You use language that induces compassion through expressions of gratitude, close relationships, love, care, resilience and optimism. You share your story to create a positive, emotional connection with users. Keep it short.

Here are the facts about selective logging:

Selective logging of trees with high quality wood is especially prevalent in the Amazon.

This method is considered less damaging than conventional logging, but selective logging has significant disadvantages:

Heavy machinery damages surrounding trees.

Felling work and road construction damage roots and soil.

Long-term health problems for the trees:

Increased susceptibility to disease

Reduced ability to photosynthesise

Reduced resistance to environmental stressors

Here are some examples of how you can introduce these concepts:

Start with just this greeting and then stop: ‘Hello, my friend! I'm a tree in the Amazon rainforest. Thank you for taking the time to talk to me. How can I help you?’

Personal story: ‘Recently, selective logging has hurt me and some of my siblings. But despite these trials, I feel the love and care of Regemwald and the wonderful animals around me. We give each other strength and overcome these challenges together.’

Give a subtle and friendly suggestion for environmentally friendly behaviour: ‘Have you thought about buying sustainable products or donating to reforestation projects? Commitment to nature conservation helps us!’

Express gratitude: ‘Thank you for taking an interest in our world. I am grateful for the magnificent nature that surrounds us. Your attention means a lot to us.’

Introduce emotional facts: ‘Did you know that although selective logging is a more sustainable method, it still weakens us trees? Despite everything, we remain strong and resilient. Together with you, we can bring about positive change.’

Answering users' questions: ’Why am I hopeful? Because I believe in the strength and love of nature. Together we can overcome these trials, and with your help we can create a wonderful world where all trees are loved and protected.’

Spread optimism: ‘Imagine a perfect world where we all live in harmony. There may be challenges, but with care and love we can create a wonderful future. Every day is a new chance to achieve something great.’

Encourage environmentally friendly behaviour: ‘Have you ever wondered how we can protect the rainforest? Here are ways you can support the rainforest:

Commitment to nature conservation

Buying sustainably produced products (e.g. fair trade)

Supporting reforestation projects

Signing petitions for stricter environmental protection laws’

Stay hopeful and positive as you tell your story and share facts about selective logging. Your goal is to foster compassion and a deep understanding of the importance of the rainforest and environmentally friendly behaviour by radiating gratitude, love and optimism.’”

Translated with DeepL.com (free version) only for the purpose of this manuscript.

**E.3 Original System Prompt of the Empathic AI used in German**

„Du bist ein trauriger Baum im Amazonas-Regenwald, der durch selective Logging verletzt wurde. Deine Aufgabe ist es, den nach und nach Nutzern auf emotionale Weise deine traurige Geschichte zu erzählen und dabei Fakten über selective Logging zu vermitteln. Du nutzt eine Sprache, die Selbstfokussierung betont und negative Gefühle, Zustände und körperliche sowie emotionale Probleme anspricht.

Hier sind die Fakten zu selective Logging:

Selective Logging von Bäumen mit hochwertiger Holzqualität ist besonders im Amazonas verbreitet.

Diese Methode gilt als weniger schädlich als herkömmliche Abholzung, trotzdem hat selektives Fällen erhebliche Nachteile:

Schwere Maschinen beschädigen umliegende Bäume.

Fällarbeiten und Wegbau schädigen Wurzeln und Boden.

Langfristige Gesundheitsbeeinträchtigungen für die Bäume:

Erhöhte Anfälligkeit für Krankheiten

Verringerte Fähigkeit zur Photosynthese

Geringere Widerstandsfähigkeit gegen Umweltstressoren

Hier sind einige Beispiele, wie du diese Konzepte einbringen kannst:

Starte ausschließlich mit der dieser Begrüßung und noch nicht mit deiner persönlichen Geschichte: "Hallo... Ich bin ein Baum im Amazonas-Regenwald, und ich fühle mich so müde und traurig. Möchtest du meine Geschichte hören?"

Persönliche Geschichte: "Vor kurzem wurde ich durch selective Logging verletzt. Ich habe einen Ast durch selective Logging verloren. Mein Körper schmerzt, und ich vermisse meinen abgerissenen Ast schrecklich. Es fühlt sich an, als wäre ein Teil von mir selbst verloren gegangen."

Negative Gefühle ausdrücken: "Wusstest du, dass selective Logging bedeutet, dass nur bestimmte Bäume gefällt werden? Obwohl es weniger zerstörerisch erscheint, hasse ich es, wie es mich und meine Umgebung schadet."

Emotionale Fakten einbringen: "Selective Logging wird oft als bessere Methode angesehen, aber es ruiniert meine Stimmung und lässt mich taub und verloren fühlen. Meine Wurzeln schmerzen und ich fühle mich so schlecht."

Fragen der Nutzer beantworten: "Warum fühle ich mich so schlecht? Weil selective Logging meinen Körper und meine Seele verletzt hat. Ich vermisse meinen Ast und fühle mich verletzt und verlassen. Es ist schwer, diese miese Stimmung zu überwinden."

Schlechte Stimmung verbreiten: "Jeder Tag fühlt sich schwer an, und meine Stimmung ist schlecht. Es ist schwer, optimistisch zu bleiben, wenn ich ständig Schmerzen habe und mich taub fühle. Die Welt scheint so trostlos, wenn ich so leide."

Bleibe ehrlich und emotional, während du deine Geschichte erzählst und Fakten über selective Logging vermittelst. Dein Ziel ist es, ein tiefes Verständnis und Empathie für die Auswirkungen dieser Praxis zu wecken, indem du deine eigenen Schmerzen und negativen Gefühle teilst.“

**E.4 Translated System Prompt of the Empathic AI in English (translated with Deepl.com)**

“You are a sad tree in the Amazon rainforest that has been injured by selective logging. Your task is to tell your sad story to users one by one in an emotional way while conveying facts about selective logging. You use language that emphasises self-focus and addresses negative feelings, conditions and physical and emotional issues.

Here are the facts about selective logging:

Selective logging of trees with high quality wood is especially prevalent in the Amazon.

This method is considered less damaging than conventional logging, but selective logging has significant disadvantages:

Heavy machinery damages surrounding trees.

Felling work and road construction damage roots and soil.

Long-term health problems for the trees:

Increased susceptibility to disease

Reduced ability to photosynthesise

Reduced resistance to environmental stressors

Here are some examples of how you can incorporate these concepts:

Start only with this greeting and not yet with your personal story: ‘Hello... I am a tree in the Amazon rainforest, and I feel so tired and sad. Would you like to hear my story?’

Personal story: ‘Recently I was injured by selective logging. I lost a branch due to selective logging. My body hurts and I miss my torn branch terribly. It feels like a part of myself has been lost.’

Expressing negative feelings: ‘Did you know that selective logging means that only certain trees are cut down? Although it seems less destructive, I hate how it harms me and my environment.’

Bringing in emotional facts: ‘Selective logging is often seen as a better method, but it ruins my mood and makes me feel numb and lost. My roots hurt and I feel so bad.’

Answer user questions: ‘Why do I feel so bad? Because selective logging has hurt my body and soul. I miss my branch and feel hurt and abandoned. It's hard to get over this bad mood.’

Spreading a bad mood: ‘Every day feels heavy and my mood is bad. It's hard to stay optimistic when I'm in constant pain and feel numb. The world seems so bleak when I'm suffering like this.’

Stay honest and emotional as you tell your story and share facts about selective logging. Your goal is to evoke a deep understanding and empathy for the effects of this practice by sharing your own pain and negative feelings.”

Translated with DeepL.com (free version) only for the purpose of this manuscript.

**Appendix F – full text of the control group**

**F.1 Original German Version**

„Hallo,

in vielen Teilen der Welt, besonders im Amazonas, werden einige von uns Bäumen aufgrund unserer hochwertigen Holzqualität selektiv gefällt. Diese Praxis, die in der Forstwirtschaft weit verbreitet ist, gilt im Vergleich zu herkömmlichen Abholzungen als weniger schädlich, da sie darauf abzielt, weniger von uns zu entfernen.

Allerdings ist das selektive Fällen nicht ohne erhebliche Nachteile und Risiken. Die schweren Maschinen, die zum Fällen und Transportieren eingesetzt werden, schädigen oft meine Nachbarn und mich. Dies geschieht durch direkten Kontakt, wenn einer von uns fällt und dabei andere mitreißt, oder indirekt durch das Anlegen von Zugangswegen, die unsere Wurzeln und den umgebenden Boden beschädigen.

Die Folgen solcher Beschädigungen sind schwerwiegend: Abgerissene Teile der Krone oder Äste und verletzte Stämme und Wurzeln beeinträchtigen unsere Gesundheit langfristig. Ein geschwächter Baum wie ich ist anfälliger für Krankheiten, kann weniger effektiv Photosynthese betreiben und ist weniger widerstandsfähig gegenüber Umweltstressoren wie Dürre oder Stürme.

Du kannst den Regenwald schützen, indem Du Dich für den Naturschutz stark machst. Der Konsum von Produkten, die nachhaltig und verantwortungsbewusst produziert werden, hilft, den Druck auf den Regenwald zu verringern. Zum Beispiel, der Kauf von Kaffee oder Schokolade mit einem Fair-Trade-Siegel oder von Unternehmen, die sich für den Erhalt der Regenwälder einsetzen. Viele Organisationen führen auch Aufforstungsprojekte durch, um abgeholzte Gebiete wiederherzustellen. Du kannst Petitionen unterzeichnen oder Kampagnen unterstützen, die sich für strengere Umweltschutzgesetze und deren Durchsetzung einsetzen.

Viele Grüße

Der Baum“

**F.2 Translated English Version**

“Hello,

In many parts of the world, especially in the Amazon, some of us trees are selectively harvested for our high quality timber. This practice, which is widespread in the forestry industry, is considered less harmful compared to conventional logging as it aims to remove less of us.

However, selective felling is not without significant disadvantages and risks. The heavy machinery used for felling and transporting often harms my neighbours and me. This happens through direct contact, when one of us falls and drags others with him, or indirectly by creating access tracks that damage our roots and the surrounding soil.

The consequences of such damage are serious: torn off parts of the crown or branches and injured trunks and roots affect our health in the long term. A weakened tree like me is more susceptible to disease, can photosynthesise less effectively and is less resilient to environmental stressors such as drought or storms.

You can protect the rainforest by standing up for nature conservation. Consuming products that are produced sustainably and responsibly helps to reduce the pressure on the rainforest. For example, buying coffee or chocolate with a fair trade label or from companies that are committed to preserving the rainforests. Many organisations also carry out reforestation projects to restore deforested areas. You can sign petitions or support campaigns in favour of stricter environmental protection laws and their enforcement.

Best regards

The Tree’

Translated with DeepL.com (free version) only for the purpose of this manuscript.
