# Supplementary figures and images for: Chatting with an LLM-based AI elicits affective and cognitive processes in education for sustainable development
Source: Sci Rep. 2026 Feb 21;16:7470. doi: 10.1038/s41598-026-39317-6 (PMC12929621; doi:10.1038/s41598-026-39317-6)

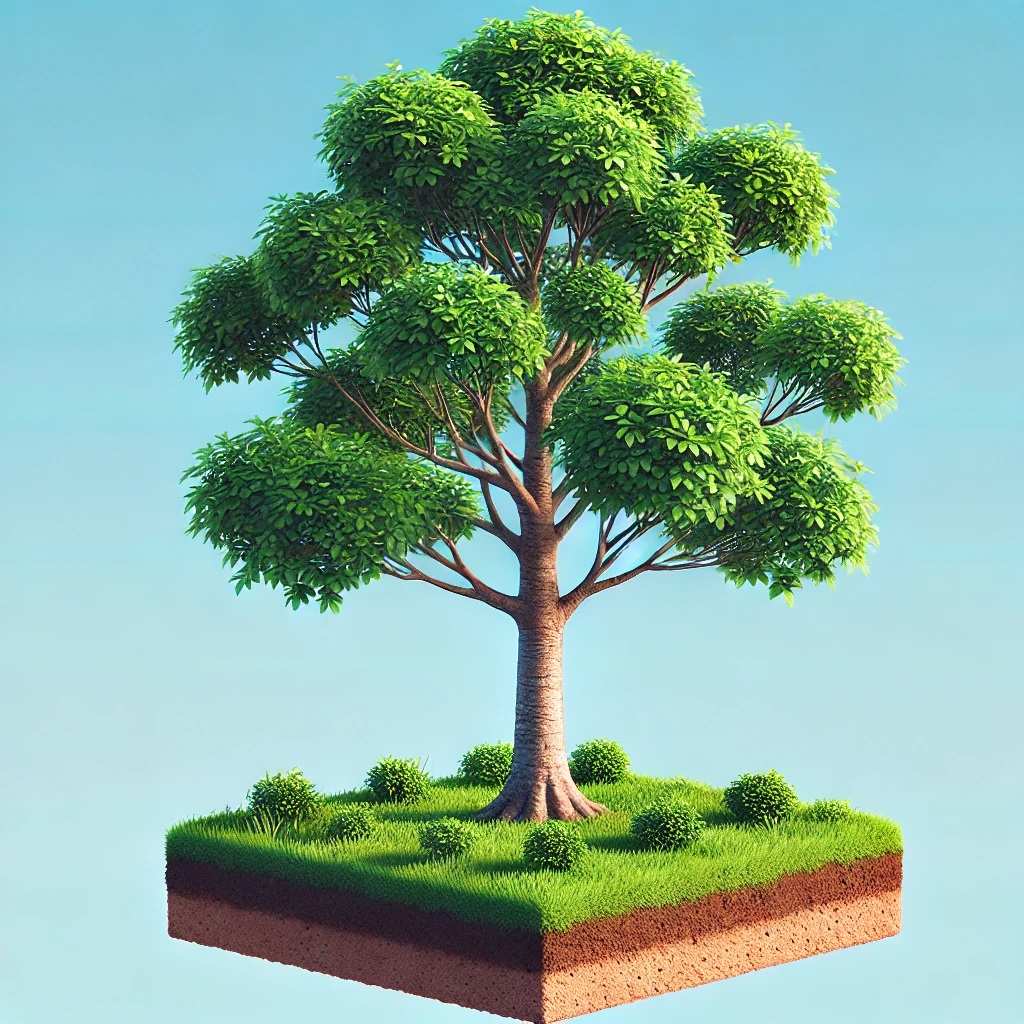

Supplement: Supplementary file 2 — Supplementary Material 2 [file 41598_2026_39317_MOESM2_ESM.zip › chatbot.jpg]

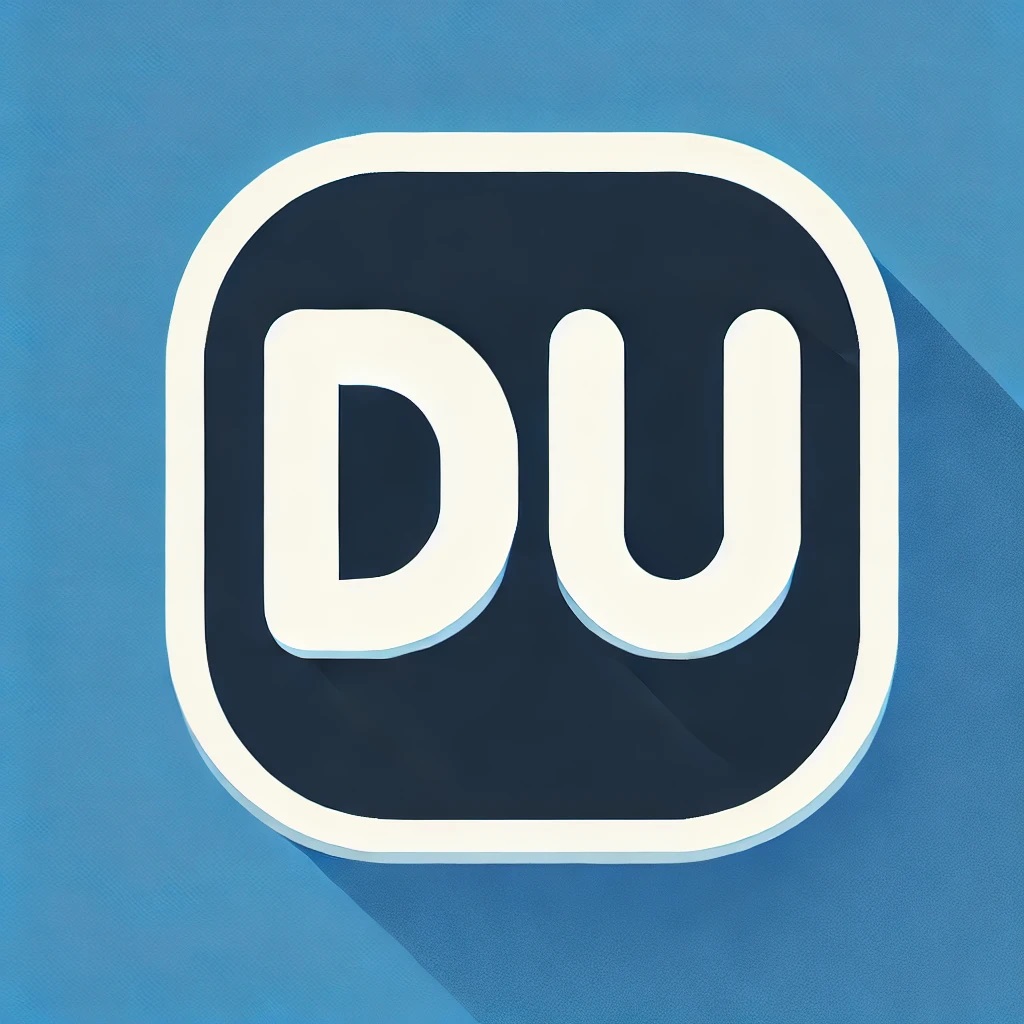

Supplement: Supplementary file 2 — Supplementary Material 2 [file 41598_2026_39317_MOESM2_ESM.zip › user.jpg]
