# Supplementary material for: Chatting with an LLM-based AI elicits affective and cognitive processes in education for sustainable development
Source: Sci Rep. 2026 Feb 21;16:7470. doi: 10.1038/s41598-026-39317-6 (PMC12929621; doi:10.1038/s41598-026-39317-6)
Supplement: Supplementary file 3 — Supplementary Material 3 [file 41598_2026_39317_MOESM3_ESM.docx]

**Applied Scoring Schemes**

1. **Scoring Scheme to assess critical reflection:**

The original was applied in German

**Instructions for coders:**

Three categories:

- Category 1: General feeling = 0 points
- Category 2: Human/nature relationship reflected in relation to one’s own situation = 1 point
- Category 3: Human/nature relationship reflected in relation to society in general = 2 points

The categories were formed based on the levels of reflection according to Smith & Hatton (1995):

**Descriptive reflection (category 1)** = All statements that provide a reason for an action or statement, including personal opinions.

**Dialogic Reflection** **(category 2)** = Discussion with oneself, examining the pros and cons of the situation / Discussion of emotions triggered.

**Critical Reflection (category 3)** = Statements that indicate that the concrete level of the event is being left behind and fundamental ethical, moral, and social problems are being considered: e.g., we must treat the forest with more respect; it is terrible what people are doing to the forest.

**Source:** Hatton, N. & Smith, D. (1995). Reflection in teacher education: Towards definition and implementation. *Teaching and Teacher Education*, *11*(1), 33–49. <https://doi.org/10.1016/0742-051X(94)00012-U>

1. **Scoring Scheme to assess changes in knowledge**

The original was applied in German

**Instructions for coders:**

**Question 1: What is meant by "selective logging"?**

- 0 points = Blank field / Don't know / No idea / “Wrong” answers (e.g., cutting down diseased trees; cutting down trees because they are in the way/blocking light, etc.)
- 1 point = statements such as “selective felling of trees instead of clearing”, “targeted harvesting of trees for commercial use”, “felling only certain trees instead of the entire rainforest”, "felling individual trees instead of clearing

**Question 2: What kind of damage do the machines mainly cause to the surrounding trees during selective felling?**

- 0 points = Blank field / Don't know / No idea / General assumptions / Vague statements
- 1 point = 1 type of damage mentioned
- 2 points = 2 types of damage mentioned
- 3 points = 3 or more types of damage mentioned

Possible damages:

- Damaged roots/soil
- Torn-off parts of the crown or branches
- Damaged trunks and roots of other trees
- More susceptible to disease
- Less effective photosynthesis
- Less resistant to environmental stressors such as drought or storms
- Less biodiversity/habitat endangered, forest as a whole endangered

**Question 3: How can the negative effects of selective felling on surrounding trees and the environment be minimized?**

- 0 points = Blank field / Don't know / No idea
- 1 point = 1 solution/suggestion given
- 2 points = 2 solutions/suggestions given
- 3 points = 3 or more solutions/suggestions given

Possible solutions:

- Promote nature conservation
- Consume products that are produced sustainably and responsibly.
- Look for quality seals (e.g., buy coffee or chocolate with a Fair Trade seal).
- Live in a resource-conscious manner.
- Support reforestation projects
- Sign petitions or support campaigns that advocate for stricter environmental protection laws and their enforcement.
